# A Wild Horse Optimization algorithm with chaotic inertia weights and its application in linear antenna array synthesis

**DOI:** 10.1371/journal.pone.0304971

**Published:** 2024-07-05

**Authors:** WanRu Zhao, Yan Liu, JianHui Li, TianNing Zhu, KunXia Zhao, Kui Hu

**Affiliations:** School of Physics and Electronic Information, Yunnan Normal University, Kunming, Yunnan Province, China; University of Lagos Faculty of Engineering, NIGERIA

## Abstract

Antennas play a crucial role in designing an efficient communication system. However, reducing the maximum sidelobe level (SLL) of the beam pattern is a crucial challenge in antenna arrays. Pattern synthesis in smart antennas is a major area of research because of its widespread application across various radar and communication systems. This paper presents an effective technique to minimize the SLL and thus improve the radiation pattern of the linear antenna array (LAA) using the chaotic inertia-weighted Wild Horse optimization (IERWHO) algorithm. The wild horse optimizer (WHO) is a new metaheuristic algorithm based on the social behavior of wild horses. The IERWHO algorithm is an improved Wild Horse optimization (WHO) algorithm that combines the concepts of chaotic sequence factor, nonlinear factor, and inertia weights factor. In this paper, the method is applied for the first time in antenna array synthesis by optimizing parameters such as inter-element spacing and excitation to minimize the SLL while keeping other constraints within the boundary limits, while ensuring that the performance is not affected. For performance evaluation, the simulation tests include 12 benchmark test functions and 12 test functions to verify the effectiveness of the improvement strategies. According to the encouraging research results in this paper, the IERWHO algorithm proposed has a place in the field of optimization.

## 1. Introduction

Antennas have long been crucial in the domain of wireless communication, extensively employed in applications such as radar systems, signal processing, and telecommunications. In today’s drive towards a technologically advanced world, array antennas have emerged as a key focus in many technology-driven fields due to their high gains and spectral efficiency [1]. These antennas are notably equipped with adaptive beamforming and beam steering capabilities [2], making them highly valuable in satellite communications. Mobile, wireless, satellite, and radar communication systems have extensively adopted antenna arrays to augment signal quality, thereby improving the system’s coverage, capacity, and link quality. The antenna array design plays a significant role in the performance of these systems [3]. Linear antenna array (LAA) use optimized excitation amplitude and phase to maintain uniform spacing with conventional arrays, or the spacing of the array elements can be optimized to maintain uniform amplitude and phase excitation for SLL minimization and null placement [4]. However, the application of mathematical tools called optimization techniques is the most challenging task in solving these problems. The antenna array problem was successfully solved by researchers who were fascinated by the encouraging results of various nature-inspired meta-heuristic optimization algorithms and achieved enhanced performance [5]. Metaheuristic optimization families are some algorithms that achieve better results in LAA synthesis, including firefly algorithm (FA) [6], cat swarm optimization (CSO) [7], biogeography-based optimization (BBO) [8], cuckoo optimization algorithm (COA) [9], genetic algorithm (GA) [10], spider monkey optimization (SMO) [11], backtracking search algorithm (BSA) [12], Taguchi method (TM) [13], differential evolution (DE) [14], colony optimization (ACO) [15], harmony search (HS) [16], bacterial foraging optimization (BFO) [17], grey wolf optimization (GWO) [18], invasive weed optimization (IWO) [19], particle swarm optimization (PSO) [20], artificial bee colony (ABC) [21], antlion optimization (ALO) [22], and much more. Recently some new optimization algorithms such as the equilibrium optimization algorithm (EOA) [23], cuckoo search algorithm (CS) [24], and mayfly algorithm (MA) [25] have also been applied with LAA synthesis. Some new results, such as enhanced firefly algorithm (EFA) [26], Teaching quality evaluation-based differential evolution (TQEDE) [27] and DNN-based Machine-Learning Method (D-DNN) [28], have been obtained by a few algorithms that were based on original contributions made earlier.

In addition, in the study by Durmus, Kurban, and Karakose (2021) [29], different swarm-based optimization algorithms are compared for their effectiveness in synthesizing linear antenna arrays, providing valuable insights into the application of these algorithms. Sharma (2023) [30] offers a comprehensive review of the use of metaheuristic algorithms in antenna array pattern synthesis, highlighting the advancements and methodologies within this area. Jafar Ramadhan Mohammed’s research advances antenna array designs crucial for 5G networks. His studies introduce methods like clustered subarray rings and phased sub-arrays to optimize antenna performance by reducing sidelobes and enhancing directivity [31, 32]. Another technique involves perturbing element excitations to form cancellation patterns, significantly suppressing sidelobes with minimal directivity loss, suitable for large arrays [33]. These innovations promise efficient, high-performance arrays for future communications. Moreover, significant progress has been made recently in applying optimization algorithms to other areas. Khettabi et al. (2022) [34] focus on sustainable process planning within reconfigurable manufacturing environments, comparing an adapted dynamic Non-dominated Sorting Genetic Algorithm II (NSGA-II) against a new NSGA-III. Kavitha et al. (2023) [35] introduce an improved Honey Bee Mating Optimization (HBMO) algorithm aimed at addressing generation expansion planning issues within deregulated networks. The application of Data Envelopment Analysis (DEA) in sustainable supplier selection is explored by Fotova Čiković et al. (2022) [36]. Kiani et al. (2021) [37] delve into the optimization of microwave transistors, employing Grey Wolf Optimization (GWO) algorithms for optimal characterization. Wang et al. (2022) [38] address the multiobjective fuzzy job-shop scheduling problem using a hybrid adaptive differential evolution algorithm. Recently emerging algorithms such as SCSO [39], GAO [40], and GOOSE [41] algorithms also provide new ideas for the research in this paper. In a nutshell, according to the no free lunch (NFL) [42] theorem, there is no optimal algorithm in all types of optimization problems. Therefore, it has been a hot topic in the field of EM optimization to find and study more efficient algorithms.

In this paper, a chaotic inertia-weighted version of WHO algorithm [43], called “IERWHO” algorithm, is proposed to enhance the original algorithm’s effectiveness and performance. The wild horse optimizer (WHO) is a new metaheuristic algorithm based on the social behavior of wild horses. Firstly, introduce Logistic tent chaotic mapping in the population initialization phase of the WHO algorithm, so that the generated initial population has better randomness than the general initial population. Secondly, the adaptive parameter TDR has been improved to enhance the ability to balance exploration and exploitation. Finally, the random inertia weight of the PSO algorithm is introduced into the water competition mechanism of the WHO algorithm to enhance and balance its exploration and development capabilities. More importantly, a series of 12 benchmark functions have been employed, alongside simulations of CEC2022 test functions, to conduct a comparative analysis of optimization efficacy within the WHO framework. This investigation elucidates the superior capabilities of the IERWHO algorithm relative to other algorithms of swarm intelligence. The IERWHO algorithm has excellent performance in solving optimization problems, so we apply it for the first time to linear antenna arrays.

The organization of this article is as follows: Section 2 presents an overview of the Wild Horse Optimization (WHO) process. Section 3 describes the utilization of the Logistic-tent sequence for the population initialization in the context of the WHO algorithm. Improvements have been made to the adaptive parameter TDR. The random inertia weight of the PSO algorithm is incorporated into the water competition mechanism of the WHO algorithm to enhance and balance its exploration and development capabilities. Additionally, the algorithm flow is described. Section 4, this paper uses 12 benchmark test functions respectively and compares and analyzes the optimization performance of each strategy in the IERWHO algorithm. CEC2022 [44] test function is also used and IERWHO algorithm is compared with other swarm intelligence algorithms. Section 5, IERWHO algorithm is applied to optimize the linear antenna array problem and compared with other algorithms for testing. In the final section, the conclusion is presented. IERWHO algorithm has demonstrated significant advancements in addressing various issues compared to the basic WHO algorithm and looks forward to the future work direction.

## 2. Wild Horse Optimization

A new bioinspired algorithm, called WHO algorithm [43], was proposed in 2021 to solve optimization problems by mimicking the hierarchy and behavior of horses. The algorithm, which is inspired by the unique mating behavior of wild horses, ensures that individuals within the same family cannot mate with each other. Additionally, the algorithm takes into account the grazing behavior of horses, where they graze together in the presence of a stallion. Furthermore, the algorithm incorporates group leadership behavior, with the stallion guiding the group to a more suitable habitat. This includes utilizing the current habitat if the group is dominant or leaving the area if another group holds dominance. Finally, the selection of the stallion is based on its fitness.

### 2.1 Creating initial populations, Horse groups, and determining leaders

*N* represents the total number of individuals, while *G* represents the number of male horses. *PS* denotes the proportion of stallions (*PS* = *G*/*N*). The remaining members, which amount to (*N*-*G*), are evenly distributed among the *G* groups.

### 2.2 Grazing behavior

Eq 1 is proposed to model grazing behavior.

$${\bar{X}}_{i,G}^{j}=2Z\cos(2\pi RZ)\times \left({\text{Stallion}}^{j}-X_{i,G}^{j}\right)+{\text{Stallion}}^{j}$$ (1)

where $X_{i,G}^{j}$ denotes the current location of the foal or mare group member, Stallion^*j*^ is the position of the stallion, *R* is a random number in [–2,2], and *Z* is the adaptive mechanism calculated from Eq 2:

$$P={\vec{R}}_{1}<\text{TDR};\;IDX=(P==0);\;Z=R_{2}\Theta \;\text{IDX}+{\vec{R}}_{3}\Theta (∼\;\text{IDX})$$ (2)

where *P* is a vector consisting of 0 and 1 equal to the dimensions of the problem, ${\vec{R}}_{1}$, *R*_2_ and ${\vec{R}}_{3}$ are random vectors with uniform distribution in the range [0, 1], IDX indexes of the returned random vector ${\vec{R}}_{1}$ satisfy the condition (*P* == 0). TDR is a parameter calculated from Eq 3 that decreases linearly from 1 to 0.


$$\text{TDR}=1-\mathrm{iter}\times \left(\frac{1}{\text{maxiter}}\right)$$ (3)

### 2.3 Horse mating behavior

To simulate the mating behavior of horses, the Crossover operator of the mean type was proposed as follows:

$$\begin{array}{l} X_{G,K}^{p}=\mathrm{Crossover}\left(X_{G,i}^{q},X_{G,j}^{z}\right)\;i\neq j\neq k,p=q=end\\ Crossover=Mean \end{array}$$ (4)

### 2.4 Group leadership

The group was taken to the water hole by the group leader (stallion). The stallions fight over the water hole, and the winning group is allowed to use it while the other groups have to leave. This process of the algorithm can be represented by the following Eq 5:

$$\bar{{\text{Stallion}}_{G_{i}}}=\left\{\begin{array}{ll} 2Z\;\cos(2\pi RZ)\times \left(\text{WH}-{\text{Stallion}}_{G_{i}}\right)+\text{WH} & \;\text{if}\;R_{3}>0.5\\ 2Z\;\cos(2\pi RZ)\times \left(\text{WH}-{\text{Stallion}}_{G_{i}}\right)-\text{WH} & \;\text{if}\;R_{3}\leq 0.5 \end{array}\right\}$$ (5)

where $Stallion_{G_{i}}$ is currently in the position of the stallion in group I, WH represents the position of the water hole. $\bar{{\text{Stallion}}_{G_{i}}}$ is next in line for group I leader.

### 2.5 Exchange and selection of leaders

If the fitness value of the leader of a group is lower than that of the positional identities are swapped:

$$Stallion_{G_{i}}=\left\{\begin{array}{cc} X_{G,i} & \;\text{if}\;\cos\;t\left(X_{G,i}\right)<\cos\;t\left({\;\text{Stallion}}_{G_{i}}\right)\\ {\text{Stallion}}_{G_{i}} & \;\text{if}\;\cos\;t\left(X_{G,i}\right)>\cos\;t\left({\text{Stallion}}_{G_{i}}\right) \end{array}\right\}$$ (6)

### 2.6 Algorithm flow of WHO

**Fig 1:**
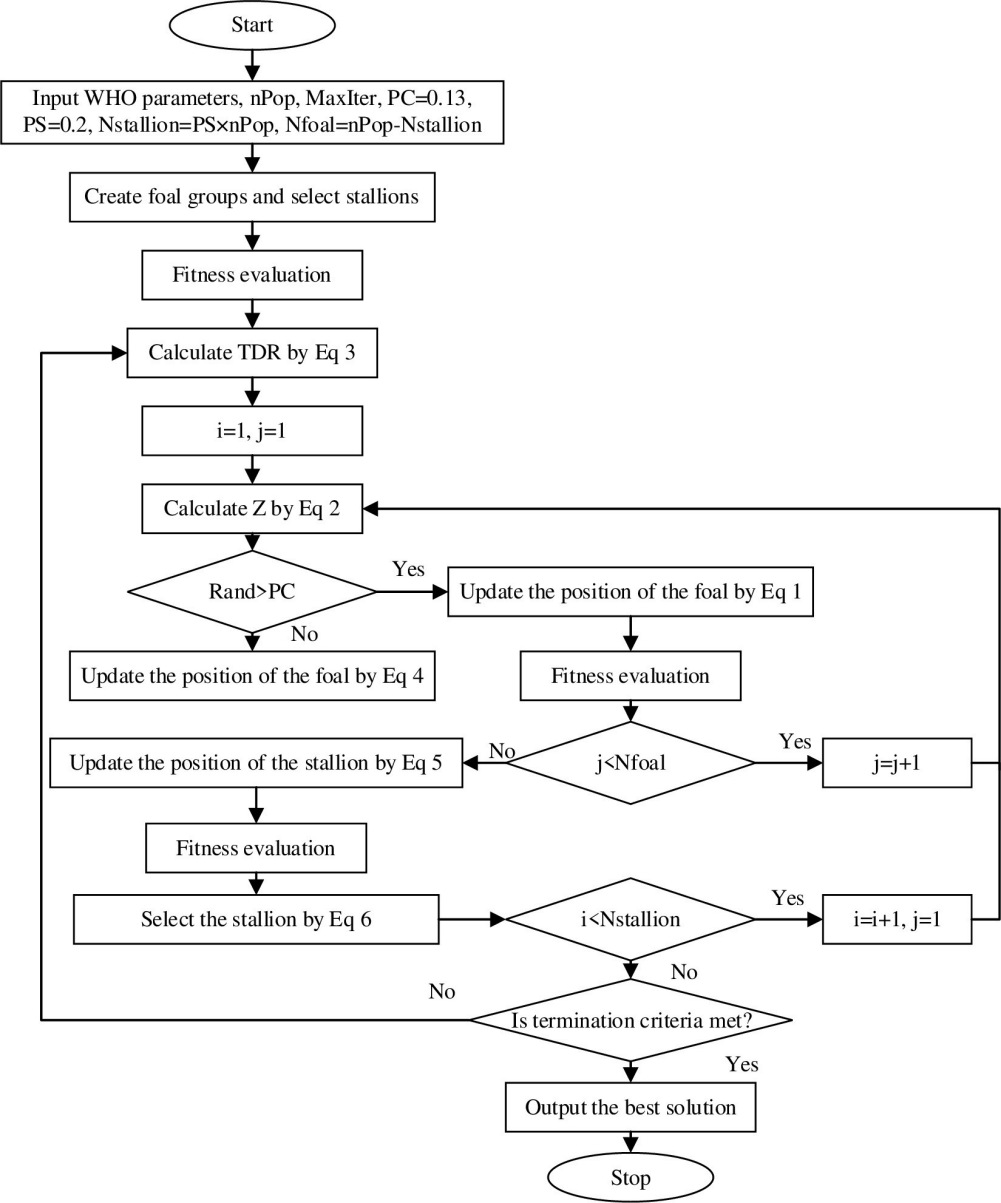
The flow chart of WHO algorithm.

The flowchart of the WHO algorithm is shown in Fig 1.

## 3. Improved Wild Horse Optimization

This section outlines three improvements to the WHO algorithm. The WHO algorithm adopts the Logistic-tent sequence initialization (IWHO), enhanced the adaptive parameter TDR (EWHO), and introduces the random inertia weight from the PSO algorithm into the WHO algorithm (RWHO). The combination of the three improvement points mentioned above is referred to as IERWHO.

### 3.1 Logistic-tent sequence initialization

**Fig 2:**
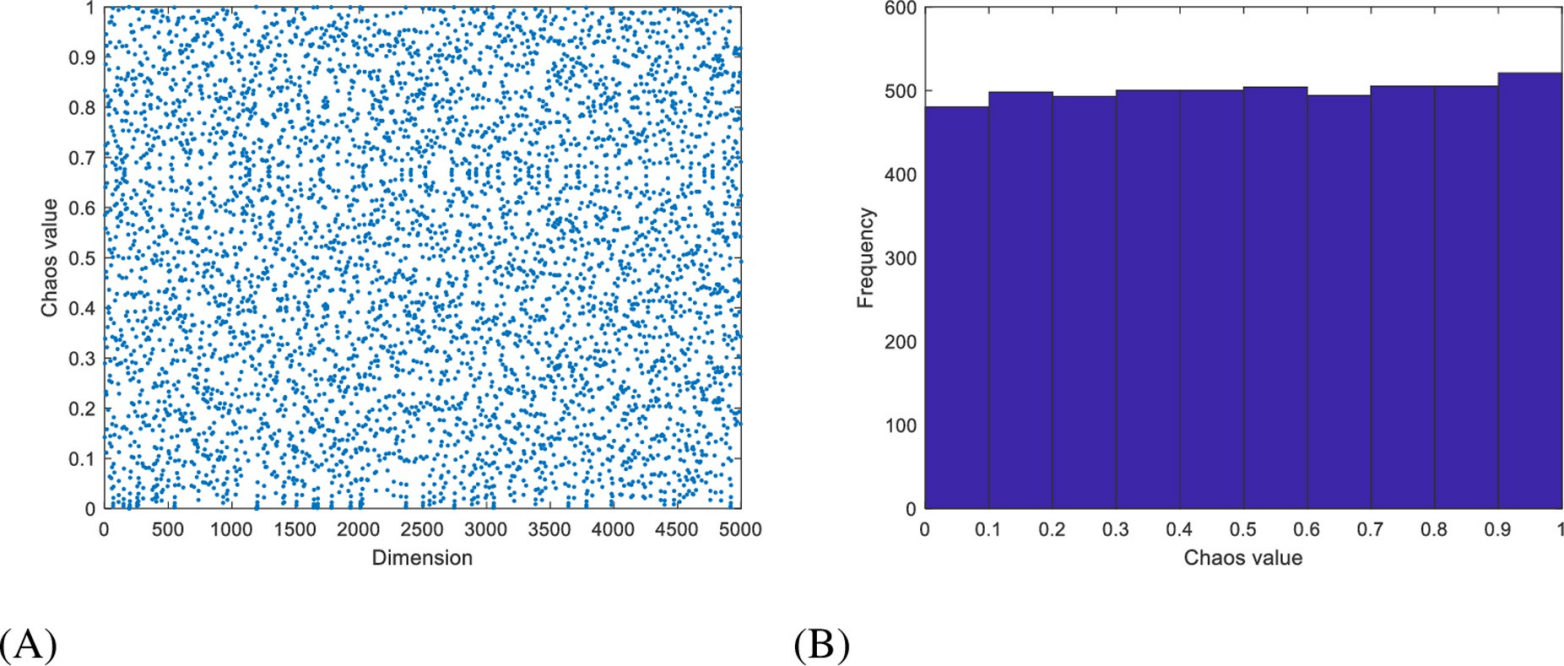
**A.** Logistic-tent chaotic mapping distribution chart. **B.** Logistic-tent chaotic mapping distribution histogram.

The algorithm’s following stages are significantly influenced by the initialization stage. The initialization of the WHO algorithm population is the random distribution of the solution space among the entire group of horses [45]. The approach exhibits low search efficiency and solution accuracy due to its high unpredictability and uneven population distribution. Furthermore, the lack of population diversity exacerbates these limitations. The Logistic-tent sequence is utilized during the population initialization stage to guide the foal population in an organized manner around the stallion, based on both individual horses’ behavioral characteristics and the random position of the stallion. Fig 2A displays the mapping distribution chart, while Fig 2B displays the map distribution histogram. The Logistic-tent mapping formula is given in Eq 7.

$$X_{n+1}=\left\{\begin{array}{l} \left[rx_{n}\left(1-x_{n}\right)+\frac{\left(4-r\right)}{2}x_{n}\right]\mathrm{mod}1,\;\text{if}\;x_{n}<0.5\\ \left[rx_{n}\left(1-x_{n}\right)+\frac{\left(4-r\right)\left(1-x_{n}\right)}{2}\right]\mathrm{mod}1,\;\text{if}\;x_{n}\geq 0.5 \end{array}\right.$$ (7)

where *X* is the system variable, *r* is the control parameter *x*_*n*_∈[0,1] and *r* ∈ [0,4].

### 3.2 Improved TDR

One of the fundamental grazing behaviors observed in the WHO algorithm involves positioning the primary horse at the heart of the grazed region, while the offspring explores its surroundings. The algorithm is still in the early stages of global search at this point. According to Eq 3, TDR is a parameter that decreases linearly from 1 to 0. The impact of this linear adaptive parameter is significantly constrained despite its capacity to achieve a harmonious balance between global exploration and local optimization. To address this issue, TDR has been modified as a nonlinear adaptive parameter. The expression is shown in Eq 8.


$$TDR={\left(1-\frac{iter}{\mathrm{maxiter}}\right)}^{{\left(\frac{iter}{\mathrm{maxiter}}\right)}^{2}}$$ (8)

**Fig 3:**
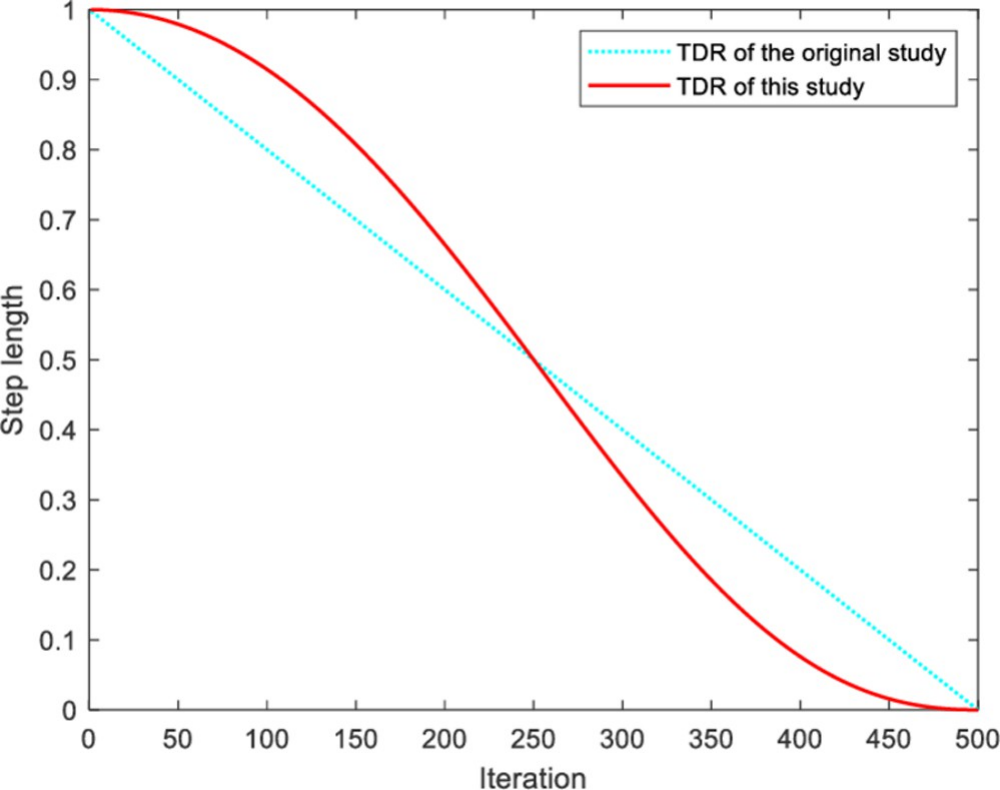
TDR curve comparison.

As shown in Fig 3, the TDR value shows a non-linear downward trend as the number of iterations increases. Increased reproductive efficiency of foals at each mating period.

### 3.3 Introduction of random inertia weights in PSO algorithm

To improve the performance of the WHO algorithm, a stochastic weight optimization strategy is proposed. Inertia weight is an important parameter in the particle velocity update formula of the PSO algorithm, which reflects the influence of previous generation particles on the current particle velocity update and is used to balance the exploration and development of the PSO algorithm [46].

This article introduces the random inertia weight of the PSO algorithm into the WHO algorithm to help the public horse find better puddles. In the initial expression of Eq 9, an extra dynamic inertia is incorporated into the puddle. The weight and the corresponding calculation formula for the inclusion of this inertia are determined as follows:

$$\omega =\omega_{\min}+(\omega_{\max}-\omega_{\min})\cdot rand\left(\right)+\sigma \cdot randn\left(\right)$$ (9)

$$\bar{{\text{Stallion}}_{G_{i}}}=\left\{\begin{array}{ll} 2Z\;\cos(2\pi RZ)\times \left(\text{WH}-{\text{Stallion}}_{G_{i}}\right)+\omega \times \text{WH} & \;\text{if}\;R_{3}>0.5\\ 2Z\;\cos(2\pi RZ)\times \left(\text{WH}-{\text{Stallion}}_{G_{i}}\right)-\omega \times \text{WH} & \text{if}\;R_{3}\leq 0.5 \end{array}\right\}$$ (10)

where *ω*_max_ and *ω*_min_ are the upper and lower boundary values, respectively, *rand* is a uniformly distributed random number between [0,1], *randn* is the random number of normal distribution. *σ* is used to measure the degree of deviation between random inertia weights and their mathematical expectations, and this term is used to control the weight error in the values.

Table 1 provides a succinct overview of the disparities between the IERWHO and WHO algorithms, as well as the motivations for enhancing these algorithms.

**Table 1:** Comparison of the WHO and IERWHO algorithms improvement points.

Function	IERWHO	WHO
improvement point 1	Logistic-tent sequence generating initial populations	Randomly generated initial populations
Formula	$X_{n+1}=\left\{\begin{array}{l} \left[rx_{n}\left(1-x_{n}\right)+\frac{\left(4-r\right)}{2}x_{n}\right]\mathrm{mod}1,\text{if}\;x_{n}<0.5\\ \left[rx_{n}\left(1-x_{n}\right)+\frac{\left(4-r\right)\left(1-x_{n}\right)}{2}\right]\mathrm{mod}1,\text{if}\;x_{n}\geq 0.5 \end{array}\right.$	$X_{n+1}=rand\left(X_{n}\right)$
Reason 1	The WHO algorithm suffers from a lack of diversity in the initial population due to the random generation of individuals. This limitation hampers the algorithm’s search flexibility. To address this, logistic-tent dual chaotic mapping methods are employed to enhance the initial population’s diversity, optimizing the global search process and increasing the algorithm’s search flexibility.	
improvement point 2	Nonlinear adaptive parameter TDR	Linear adaptive parameter TDR
Formula	$TDR={\left(1-\frac{iter}{\mathrm{maxiter}}\right)}^{{\left(\frac{iter}{\mathrm{maxiter}}\right)}^{2}}$	$\text{TDR}=1-\mathrm{iter}\times \left(\frac{1}{\text{maxiter}}\right)$
Reason 2	The impact of this linear adaptive parameter is significantly constrained despite its capacity to achieve a harmonious balance between global exploration and local optimization. To address this issue, TDR has been modified as a nonlinear adaptive parameter.	
improvement point 3	The random inertia weight of the PSO algorithm is incorporated into the water competition mechanism	Water competition mechanism
Formula	$\omega =\omega_{\min}+(\omega_{\max}-\omega_{\min})\cdot rand\left(\right)+\sigma \cdot randn\left(\right)$ $\bar{{\text{Stallion}}_{G_{i}}}=\left\{\begin{array}{ll} \begin{array}{l} 2Z\;\cos(2\pi RZ)\\ \times \left(\text{WH}-{\text{Stallion}}_{G_{i}}\right)+\omega \times \text{WH} \end{array} & \;\text{if}\;R_{3}>0.5\\ \begin{array}{l} 2Z\;\cos(2\pi RZ)\\ \times \left(\text{WH}-{\;\text{Stallion}}_{G_{i}}\right)-\omega \times \text{WH} \end{array} & \;\text{if}R_{3}\leq 0.5 \end{array}\right\}$	$\bar{{\text{Stallion}}_{G_{i}}}=\left\{\begin{array}{ll} \begin{array}{l} 2Z\;\cos(2\pi RZ)\\ \times \left(\text{WH}-{\text{Stallion}}_{G_{i}}\right)+\text{WH} \end{array} & \;\text{if}\;R_{3}>0.5\\ \begin{array}{l} 2Z\;\cos(2\pi RZ)\\ \times \left(\text{WH}-{\text{Stallion}}_{G_{i}}\right)-\text{WH} \end{array} & \;\text{if}\;R_{3}\leq 0.5 \end{array}\right\}$
Reason 3	The water resource competition mechanism of the WHO algorithm suffers from the problems of uneven water resource allocation and slow update of stallion position. For this reason, the stochastic inertia weights of the particle swarm algorithm are introduced to increase the diversity of water resources allocation, which further dynamically balances the global search ability of the regulation algorithm and accelerates the convergence of the algorithm.	

### 3.4 Algorithm flow of IERWHO

**Fig 4:**
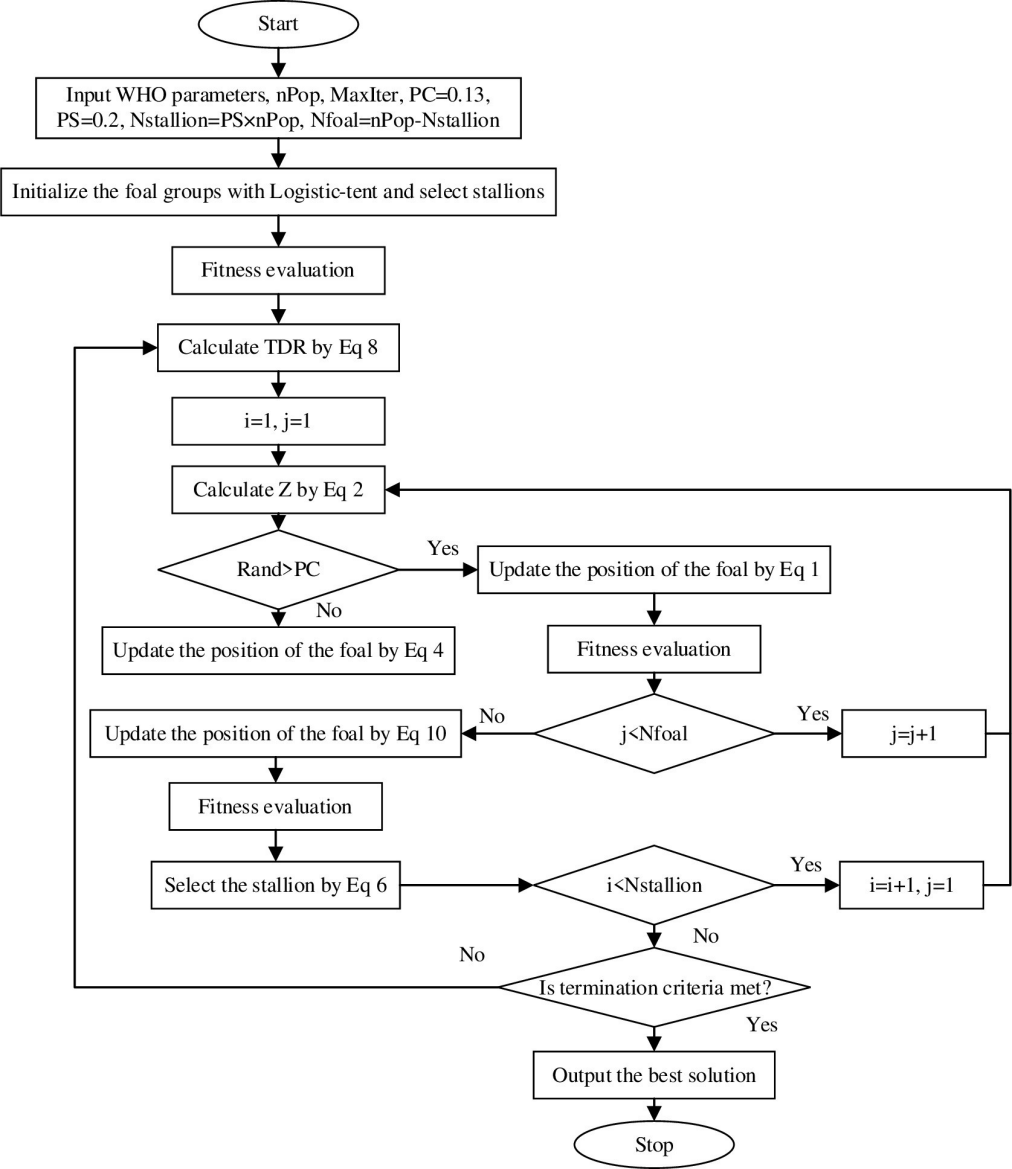
The flow chart of IERWHO algorithm.

The flowchart of the proposed IERWHO algorithm is shown in Fig 4.

## 4. Simulation experiment and result analysis

The section discusses the effectiveness analysis of the IERWHO algorithm on 12 benchmark functions, including both unimodal and multimodal tests. Different improvement strategies are introduced and compared using metrics such as best, worst, mean, and standard deviation. Results show that IERWHO outperforms the WHO algorithm across dimensions. Additionally, a comparison with different swarm intelligence algorithms on the CEC2022 benchmark demonstrates IERWHO algorithm’s competitiveness and effectiveness.

### 4.1 Effectiveness analysis of improved strategies

#### 4.1.1. Selection of test functions

The performance and optimization effect of IERWHO algorithm is evaluated by selecting 12 benchmark functions, as depicted in Table 2. Table 2 contains the specifics of the unimodal (F1-F8) and multimodal (F9-F12) test functions [43, 47]. Typically, optimization algorithms consist of two primary stages which are exploration and development. The unimodal test function does not have a local optimum, but an overall optimum. The dedication of all search spaces lies in global optimization. Hence, an important criterion to consider is the convergence speed and utilization rate of the algorithm. The evaluation of an algorithm’s performance can be carried out effectively by employing multimodal and hybrid test functions that encompass numerous local optima.

**Table 2:** Parameter sets of the chosen algorithms.

Function type	Function	Range	*f_MN_*
Unimodal functions	$f_{1}(x)=\sum_{i=1}^{n}x_{i}^{2}$	[–100, 100]	0
	$f_{2}(x)=\sum_{i=1}^{n}ix_{i}^{2}$	[–10, 10]	0
	$f_{3}(x)=\sum_{i=1}^{n}{\left(\sum_{j-1}^{i}x_{j}\right)}^{2}$	[–100, 100]	0
	$f_{4}(x)=\max\left\{|x_{i}|,1\leq i\leq n\right\}$	[–100, 100]	0
	$f_{5}(x)=\sum_{i=1}^{n}\left|x_{i}\right|+\prod_{i=1}^{n}\left|x_{i}\right|$	[–10, 10]	0
	$f_{6}(x)=\max\left\{|x_{i}|,1\leq i\leq n\right\}$	[-1.28, 1.28]	0
	$f_{7}(x)=\sum_{i=1}^{n}ix_{i}^{4}$	[-1.28,1.28]	0
	$f_{8}(x)=\sum_{i=1}^{n}{\left|x_{i}\right|}^{\left(i+1\right)}$	[–1, 1]	0
Multimodal functions	$F_{9}(x)=-20\exp\left(-0.2\sqrt{\frac{1}{n}\sum_{i=1}^{n}x_{i}^{2}}\right)-\exp\left(\frac{1}{n}\sum_{i=1}^{n}\cos\left(2\pi x_{i}\right)\right)+20+e$	[–32, 32]	0
	$F_{10}(x)=\sum_{i=1}^{n}x_{i}^{2}+{\left(\sum_{i=1}^{n}0.5ix_{i}\right)}^{2}+{\left(\sum_{i=1}^{n}0.5ix_{i}\right)}^{4}$	[–1, 1]	0
	$F_{11}(x)=\sum_{i=1}^{n}{\left(10^{6}\right)}^{\left(i-1)/(n-1\right)}x_{i}^{2}$	[–10, 10]	0
	$F_{12}(x)=\sum_{i=1}^{n}\left|x_{i}\cdot \sin\left(x_{i}\right)+0.1\cdot x_{i}\right|$	[–10, 10]	0

#### 4.1.2. Effectiveness analysis of different improvement strategies

In order to enhance the assessment of the enhanced strategies in the IERWHO algorithm, four algorithms have been put forward. These algorithms aim to improve the verification process by evaluating the efficacy of each strategy. The WHO algorithm adopts the Logistic-tent sequence initialization (IWHO), enhanced the adaptive parameter TDR (EWHO), and introduces the random inertia weight from the PSO algorithm into the WHO algorithm (RWHO). Table 3 includes a detailed comparison of the unique features of the IERWHO, IWHO, EWHO, RWHO and WHO algorithms. Such a Table 3 would serve as an informative tool for understanding the different characteristics and applications of each WHO algorithm, providing a clear comparison of their functionalities and use cases. In this study, the performance of several evolutionary algorithms, including WHO, IWHO, EWHO, RWHO, and IERWHO algorithms, was examined on a set of 12 benchmark test functions. In order to assess IERWHO algorithm’s optimization ability, the search space dimensions d of F1-F12 were set to 30, respectively. The other parameters were set in a uniform way as follows: population size *N* = 30, maximum iteration *T*_max_ = 500; and the parameters in the algorithm *PC* = 0.13, *PS* = 0.2.

**Table 3:** Succinctly outlines the disparities between IERWHO, IWHO, EWHO, RWHO and WHO algorithms.

Function	WHO	IWHO	EWHO	RWHO	IERWHO
Creating initial populations	Randomly generated initial populations	Logistic-tent sequence generating initial populations	Randomly generated initial populations	Randomly generated initial populations	Logistic-tent sequence generating initial populations
Adaptive parameters TDR	Linear adaptive parameter TDR	Linear adaptive parameter TDR	Nonlinear adaptive parameter TDR	Linear adaptive parameter TDR	Nonlinear adaptive parameter TDR
Exchange and selection of leaders	Water competition mechanism	Water competition mechanism	Water competition mechanism	The random inertia weight is incorporated into the water competition mechanism	The random inertia weight is incorporated into the water competition mechanism

Statistical results were obtained by solving each test function 30 times. Various performance metrics, such as best, worst, mean, and standard deviation, were used to compare algorithms. The iterations produced the best solutions. It is important that the values of standard deviation and mean deviation are minimized. These values indicate the algorithm’s effectiveness in avoiding local optimality and achieving the global optimal solution.

The statistics in Table 4 shows that, under the 30 dimensions condition, IERWHO algorithm has a much better best value, worst value, average value, and standard deviation than WHO algorithm. The proposed IERWHO algorithm method produces better results than existing methods on all unimodal detection functions, and IWHO, EWHO, and IERWHO algorithms also have different magnitudes of improvement compared to WHO algorithm. The values obtained by IWHO algorithm for the F4 and F6 functions were found to be slightly inferior to those determined by WHO algorithm. However, the discrepancy is minimal and falls within an acceptable range. Both sets of values are considered to be reliable and valid. For all multimodal test functions, the proposed IERWHO algorithm method yields better results than existing methods. Especially, RWHO and IERWHO algorithms achieved theoretical optimal values in each iteration of the F9-F12 function, while IWHO and EWHO algorithms also improved compared to WHO algorithm at different functions.

**Table 4:** Optimization results of test functions for different algorithm improvement points.

Function		WHO	IWHO	EWHO	RWHO	IERWHO
F1	Best	1.9277E-52	3.6847E-51	3.0045E-179	0.0000E+00	**0.0000E+00**
	Worst	2.1286E-42	2.8072E-43	1.8166E-162	4.8444E-301	**0.0000E+00**
	Mean	9.8761E-44	2.1785E-44	6.1081E-164	1.6846E-302	**0.0000E+00**
	Std	3.9839E-43	6.327E-44	0.0000E+00	0.0000E+00	**0.0000E+00**
F2	Best	4.2507E-52	6.2774E-55	4.8061E-160	0.0000E+00	**0.0000E+00**
	Worst	3.3213E-42	3.8504E-42	2.6414E-137	3.5734E-303	**0.0000E+00**
	Mean	1.1496E-43	1.4875E-43	1.0813E-138	1.2627E-304	**0.0000E+00**
	Std	6.0588E-43	7.0584E-43	4.8425E-138	0.0000E+00	**0.0000E+00**
F3	Best	4.8045E-34	1.0117E-36	1.9419E-171	0.0000E+00	**0.0000E+00**
	Worst	1.9831E-24	2.4063E-23	1.5152E-146	6.7891E-282	**0.0000E+00**
	Mean	1.3958E-25	8.0748E-25	5.0508E-148	2.263E-283	**0.0000E+00**
	Std	3.9501E-25	4.3922E-24	2.7664E-147	0.0000E+00	**0.0000E+00**
F4	Best	2.5711E-20	7.8303E-20	1.1412E-87	3.4715E-176	**1.009E-196**
	Worst	6.5012E-17	3.607E-16	1.4333E-76	7.8258E-148	**2.1671E-179**
	Mean	1.0549E-17	3.997E-17	4.7872E-78	2.6086E-149	**7.8202E-181**
	Std	1.4882E-17	7.995E-17	2.6167E-77	1.4288E-148	**0.0000E+00**
F5	Best	6.0921E-29	2.7176E-31	7.5029E-94	6.4096E-178	**1.0299E-204**
	Worst	6.848E-23	1.2187e-24	7.8086E-85	2.6874E-152	**4.3385E-181**
	Mean	2.6221E-24	1.065E-25	6.5427E-86	1.1692E-153	**2.7083E-182**
	Std	1.2467E-23	2.3926E-25	1.8955E-85	4.9971E-153	**0.0000E+00**
F6	Best	0.00031151	0.00033473	7.4446E-06	1.0021E-05	**7.3767E-06**
	Worst	0.0047092	0.0069325	0.00073668	0.00131	**0.00052089**
	Mean	0.0014407	0.0018265	0.0002259	0.00047371	**0.00016647**
	Std	0.0010223	0.0015695	0.00017943	0.00034329	**0.00015711**
F7	Best	1.2387E-97	3.4902E-105	1.1034E-313	0.0000E+00	**0.0000E+00**
	Worst	2.8399E-82	5.1438E-80	5.9856E-274	0.0000E+00	**0.0000E+00**
	Mean	1.0784E-83	2.0439E-81	2.017E-275	0.0000E+00	**0.0000E+00**
	Std	5.1837E-83	9.5006E-81	0.0000E+00	0.0000E+00	**0.0000E+00**
F8	Best	1.2387E-97	3.4902E-105	1.1034E-313	0.0000E+00	**0.0000E+00**
	Worst	2.8399E-82	5.1438E-80	5.9856E-274	0.0000E+00	**0.0000E+00**
	Mean	1.0784E-83	2.0439E-81	2.017E-275	0.0000E+00	**0.0000E+00**
	Std	5.1837E-83	9.5006E-81	0.0000E+00	0.0000E+00	**0.0000E+00**
F9	Best	0.0000E+00	0.0000E+00	0.0000E+00	0.0000E+00	**0.0000E+00**
	Worst	9.5638E-06	0.0000E+00	0.0000E+00	0.0000E+00	**0.0000E+00**
	Mean	1.5987E-15	2.0724E-15	1.125E-15	8.8818E-16	**8.8818E-16**
	Std	1.4454E-15	1.7034E-15	9.0135E-16	0.0000E+00	**0.0000E+00**
F10	Best	0.0097159	0.0097159	0.0000E+00	0.0000E+00	**0.0000E+00**
	Worst	0.037224	0.078189	0.0097159	0.0000E+00	**0.0000E+00**
	Mean	9.9658E-107	1.343E-106	2.4329E-210	0.0000E+00	**0.0000E+00**
	Std	3.8224E-106	5.5804E-106	0.0000E+00	0.0000E+00	**0.0000E+00**
F11	Best	6.0445E-118	1.4172E-112	5.0495E-236	0.0000E+00	**0.0000E+00**
	Worst	1.1305E-94	4.3571E-97	1.3081E-200	0.0000E+00	**0.0000E+00**
	Mean	1.961E-39	4.9909E-38	5.3811E-135	1.2718E-286	**0.0000E+00**
	Std	7.7941E-39	2.6649E-37	2.7924E-134	0.0000E+00	**0.0000E+00**
F12	Best	7.1823E-14	4.8987E-09	7.3963E-76	0.0000E+00	**0.0000E+00**
	Worst	0.39987	0.69987	0.099873	6.7542E-116	**0.0000E+00**
	Mean	0.0013166	0.0084599	0.0015741	2.1548E-148	**7.2959E-180**
	Std	0.0021454	0.039678	0.0042548	1.1762E-147	**0.0000E+00**

**Fig 5:**
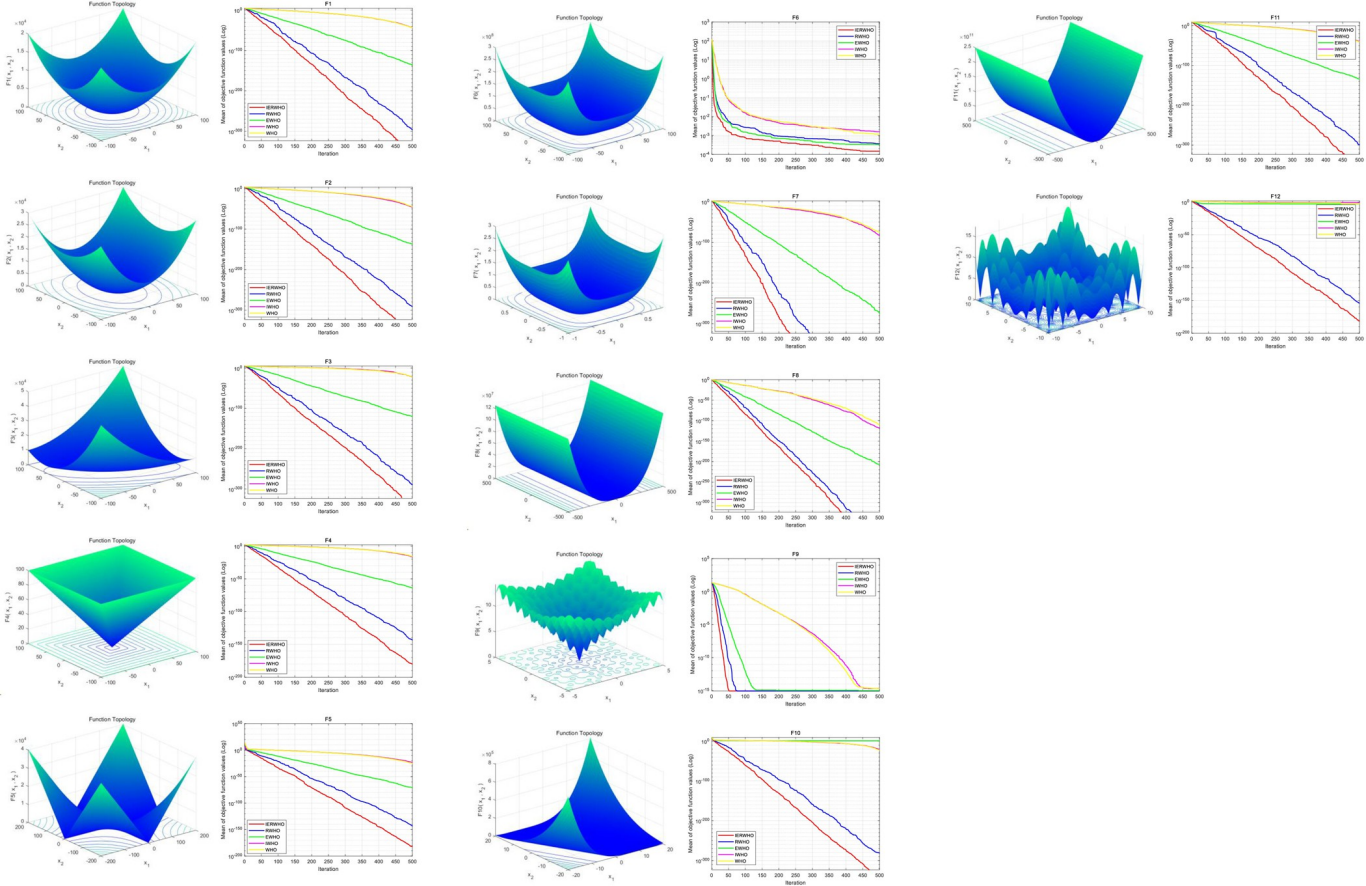
Function graph and algorithm iterative graph.

As depicted in Fig 5, the iteration rules for the functions exhibit remarkable resemblance. In F1 to F12, IERWHO demonstrates the quickest descent to the lowest objective function values, indicating superior performance in terms of both speed and ability to find a lower minimum. This performance suggests that IERWHO is particularly effective at the search space of various functions and possibly avoiding local minima. The simulation outcomes showcase the excellent performance and competitiveness of IERWHO algorithm in addressing these optimization issues.

### 4.2. Comparison between IERWHO algorithm and different swarm intelligence algorithms

**Fig 6:**
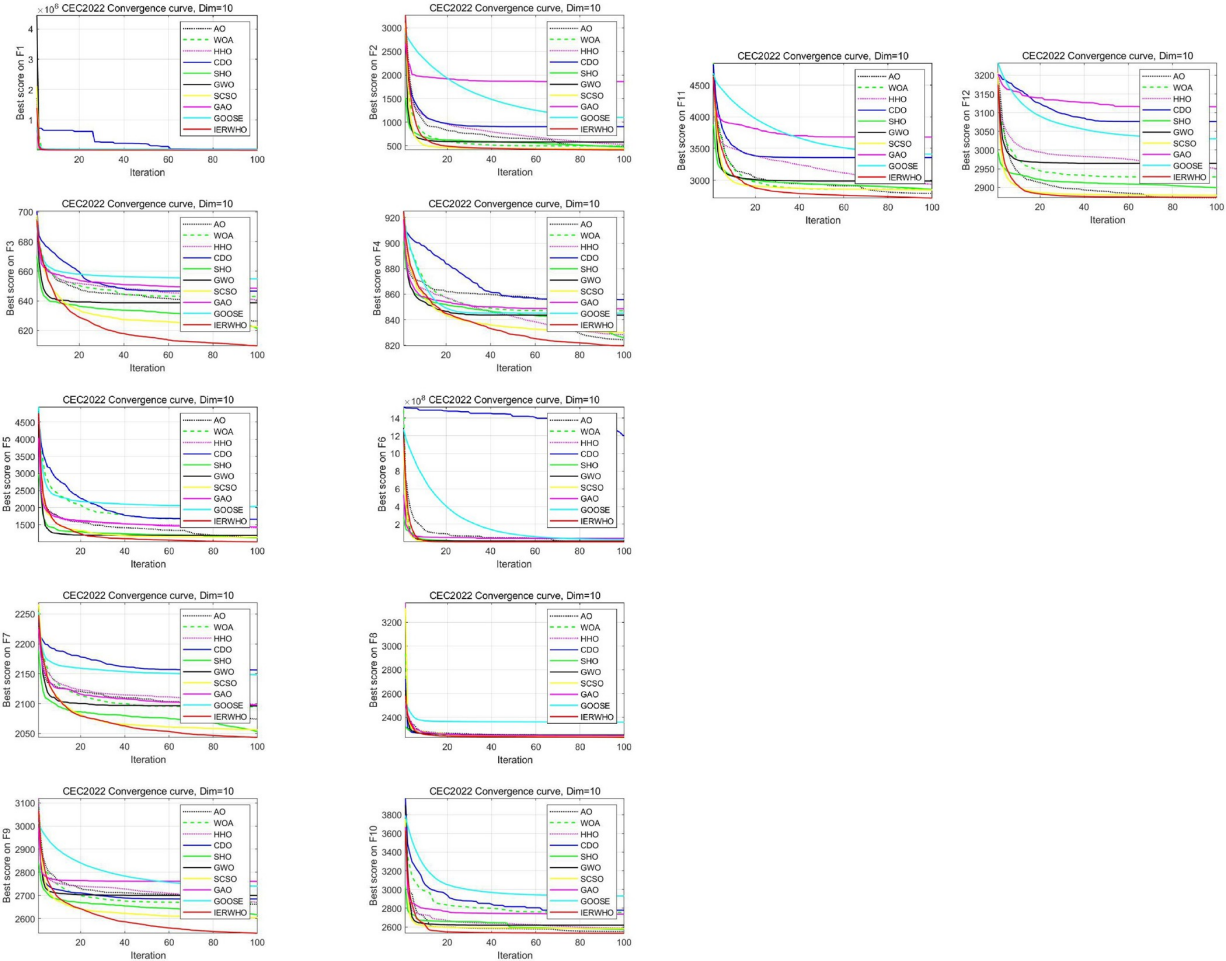
The convergence curves of all the 10 algorithms.

The IERWHO algorithm that was proposed has been tested on 12 functions of the famous CEC2022 benchmark. The parameter settings for each of the competing algorithms are listed in Table 5 for the experiments. For every benchmark function, the number of individuals is 50, the algorithm executes 100 iterations and emulates 30 runtimes, with a dimension of 10. Simply put, these benchmark functions are generally categorized into four major types: unimodal, basic, hybrid, and composition functions. In this regard, several well-known algorithms, such as WOA [48], HHO [49], AO [50], SHO [51], CDO [52], GWO [18], SCSO [39], GAO [40], and GOOSE [41], are compared with the IERWHO algorithm. Fig 6 plots the convergence curves for all six algorithms in Table 5 on the 10-dimensional problem.

**Table 5:** Controlling parameter values of the tested algorithms.

Algorithms	Parameters	Values
WOA	Whales Number	50
	*a*	∈[0,2]
	*a*2	∈[–1,–2]
HHO	Harris Hawks Number	50
	*β*	1.5
	*E*_0_	∈[–1,1]
AO	Materials	50
	*α*	0.1
	δ	0.1
	*r*_1_	10
	*U*	0.00565
	*ω*	0.005
SHO	Sea-horse Number	50
	*r*_1_	0
	probability of success *r*_2_	0.1
CDO	Elephants Number	50
	*S*_γ_	Rand (1, 300,000) km/s
	*S_β_*	Rand (1, 270,000) km/s
	*S_α_*	Rand (1, 16,000) km/s
	*r*	Rand (0, 1)
GWO	Wolf Number	50
	a	[2,0]
	A	[2,0]
	C	2.rand(0,1)
SCSO	Sand Cat Number	50
	Sensitivity range (*r_G_*)	[2,0]
	Phases control range (*R*)	[-2*r_G_*,-2*r_G_*]
GAO	Armadillo Number	50
	*r*	[0,1]
	I	1 or 2
GOOSE	Goose Number	50
	*pro*	[0,1]
	*rnd*	[0,1]
	*coe*	[0,1]
	*alpha*	[2,0]
IERWHO	Horses Number	50
	PC	0.13
	PS	0.2
	Crossover	Mean

The function F1 is unimodal, meaning they have only one global optimum. This function is commonly used to evaluate how well an algorithm can exploit its resources compared to other metaheuristics. From Table 6, it is evident that the IERWHO algorithm is highly competitive with the compared metaheuristic algorithms. Basic functions, specifically F2-F5, include various simple mathematical functions to test the fundamental performance of an algorithm, while unimodal functions are specifically used to evaluate the algorithm’s performance in situations where there is only one optimal solution. Therefore, the use of this benchmark function is highly beneficial in evaluating the exploratory capabilities of a compared metaheuristic algorithm. The IERWHO algorithm has near perfect searchability, as evidenced by the reported results of the F2-F5 functions in Table 6. The IERWHO algorithm is consistently the most effective or suboptimal in many benchmark problems. The IERWHO algorithm’s comprehensive exploration techniques drive it towards the desired globally optimal direction, which is the cause of this success. In this article, we propose the utilization of the highly complex mathematical and hybrid functions F9-F12 to assess the capabilities of the IERWHO algorithm and other competitive algorithms in terms of exploration, development, and escaping local optimal traps. Through our experimentations, as exhibited in Table 6, it is evident that the IERWHO algorithm proves to be highly effective, outperforms competitors in mean, standard, best and worst value derivation, in 10 dimensions.

**Table 6:** Experimental optimization results for dimension 10 using reported competitor algorithms on the unimodal, multimodal, hybrid, and composite of CEC2022 benchmark functions.

Function		AO	WOA	HHO	CDO	SHO	GWO	SCSO	GAO	GOOSE	IERWHO
F1	Mean	7.25E+03	3.14E+04	5.97E+03	2.92E+04	5.26E+03	9.07E+03	3.43E+03	7.75E+03	2.76E+04	**2.96E+03**
	Std	**1.45E+03**	1.26E+04	1.57E+03	5.34E+04	2.34E+03	3.54E+03	2.66E+03	1.49E+03	1.32E+04	1.50E+03
	Best	3.85E+03	7.76E+03	1.32E+03	9.81E+03	1.22E+03	3.18E+03	**3.53E+02**	4.93E+03	6.84E+03	7.88E+02
	Worst	1.08E+04	5.75E+04	8.57E+03	3.05E+05	9.28E+03	1.73E+04	9.54E+03	1.06E+04	5.43E+04	**7.15E+03**
F2	Mean	4.88E+02	4.99E+02	5.30E+02	9.05E+02	4.76E+02	5.81E+02	4.35E+02	1.86E+03	1.10E+03	**4.13E+02**
	Std	7.05E+01	8.70E+01	9.33E+01	3.25E+01	7.40E+01	7.52E+01	3.16E+01	5.79E+02	4.97E+02	**1.40E+01**
	Best	4.20E+02	4.11E+02	4.17E+02	8.49E+02	4.03E+02	4.95E+02	**4.00E+02**	7.25E+02	4.96E+02	4.00E+02
	Worst	8.32E+02	8.43E+02	8.07E+02	9.67E+02	6.88E+02	8.35E+02	4.96E+02	3.14E+03	2.55E+03	**4.75E+02**
F3	Mean	6.26E+02	6.43E+02	6.40E+02	6.47E+02	6.21E+02	6.39E+02	6.23E+02	6.49E+02	6.55E+02	**6.10E+02**
	Std	6.66E+00	1.45E+01	1.10E+01	6.50E+00	**5.90E+00**	7.44E+00	1.12E+01	8.21E+00	1.08E+01	6.18E+00
	Best	6.13E+02	6.18E+02	6.20E+02	6.35E+02	6.11E+02	6.26E+02	6.04E+02	6.31E+02	6.36E+02	**6.02E+02**
	Worst	6.39E+02	6.74E+02	6.59E+02	6.59E+02	6.34E+02	6.55E+02	6.44E+02	6.59E+02	6.76E+02	**6.28E+02**
F4	Mean	8.24E+02	8.47E+02	8.28E+02	8.56E+02	8.26E+02	8.44E+02	8.30E+02	8.49E+02	8.45E+02	**8.20E+02**
	Std	6.92E+00	1.34E+01	7.14E+00	7.78E+00	8.39E+00	7.46E+00	7.76E+00	**6.16E+00**	1.54E+01	7.40E+00
	Best	8.10E+02	8.27E+02	8.10E+02	8.43E+02	8.16E+02	8.31E+02	8.13E+02	8.36E+02	8.25E+02	**8.08E+02**
	Worst	8.39E+02	8.79E+02	8.41E+02	8.72E+02	8.55E+02	8.58E+02	8.48E+02	8.60E+02	8.92E+02	**8.38E+02**
F5	Mean	1.13E+03	1.66E+03	1.42E+03	1.66E+03	1.12E+03	1.19E+03	1.14E+03	1.44E+03	2.03E+03	**1.00E+03**
	Std	1.14E+02	5.39E+02	1.96E+02	1.11E+02	1.08E+02	1.63E+02	1.90E+02	1.37E+02	5.25E+02	**8.80E+01**
	Best	9.46E+02	9.97E+02	1.01E+03	1.40E+03	9.47E+02	9.48E+02	9.11E+02	1.19E+03	1.28E+03	**9.04E+02**
	Worst	1.39E+03	3.12E+03	1.90E+03	1.88E+03	1.35E+03	1.62E+03	1.60E+03	1.67E+03	3.16E+03	**1.21E+03**
F6	Mean	6.44E+05	5.76E+04	1.52E+04	1.20E+09	9.98E+03	6.78E+06	4.65E+03	3.84E+07	2.72E+07	**2.95E+03**
	Std	1.08E+06	1.06E+05	1.44E+04	1.03E+09	9.26E+03	5.19E+06	2.19E+03	2.84E+07	7.82E+07	**1.35E+03**
	Best	1.13E+04	3.44E+03	2.75E+03	5.74E+07	2.37E+03	2.94E+05	2.01E+03	1.02E+06	1.87E+03	**1.86E+03**
	Worst	5.70E+06	5.48E+05	6.52E+04	3.24E+09	3.87E+04	1.92E+07	9.29E+03	1.02E+08	3.65E+08	**7.04E+03**
F7	Mean	2.07E+03	2.09E+03	2.10E+03	2.16E+03	2.05E+03	2.10E+03	2.06E+03	2.10E+03	2.15E+03	**2.04E+03**
	Std	1.64E+01	3.43E+01	3.43E+01	**1.07E+01**	1.86E+01	2.12E+01	2.86E+01	1.50E+01	5.75E+01	1.39E+01
	Best	2.05E+03	2.05E+03	2.03E+03	2.13E+03	**2.03E+03**	2.06E+03	2.03E+03	2.06E+03	2.04E+03	2.03E+03
	Worst	2.12E+03	2.20E+03	2.16E+03	2.17E+03	2.10E+03	2.14E+03	2.14E+03	2.14E+03	2.27E+03	**2.08E+03**
F8	Mean	2.23E+03	2.24E+03	2.24E+03	2.24E+03	**2.23E+03**	2.25E+03	2.23E+03	2.24E+03	2.36E+03	2.23E+03
	Std	4.90E+00	1.77E+01	1.38E+01	1.25E+01	**2.93E+00**	3.02E+01	2.38E+01	2.13E+01	1.07E+02	2.06E+01
	Best	2.23E+03	2.23E+03	2.21E+03	2.23E+03	2.21E+03	2.23E+03	**2.21E+03**	2.23E+03	2.22E+03	2.22E+03
	Worst	2.24E+03	2.30E+03	2.28E+03	2.28E+03	**2.23E+03**	2.36E+03	2.36E+03	2.34E+03	2.59E+03	2.34E+03
F9	Mean	2.66E+03	2.67E+03	2.67E+03	2.68E+03	2.62E+03	2.70E+03	2.60E+03	2.76E+03	2.74E+03	**2.54E+03**
	Std	3.76E+01	4.53E+01	3.79E+01	1.45E+01	3.39E+01	2.91E+01	4.93E+01	5.32E+01	8.82E+01	**9.47E+00**
	Best	2.60E+03	2.56E+03	2.56E+03	2.67E+03	2.54E+03	2.65E+03	2.53E+03	2.64E+03	2.58E+03	**2.53E+03**
	Worst	2.72E+03	2.74E+03	2.74E+03	2.72E+03	2.69E+03	2.78E+03	2.69E+03	2.88E+03	3.01E+03	**2.57E+03**
F10	Mean	2.55E+03	2.76E+03	2.59E+03	2.78E+03	2.57E+03	2.62E+03	2.58E+03	2.74E+03	2.93E+03	**2.53E+03**
	Std	6.27E+01	4.59E+02	1.50E+02	2.24E+02	6.27E+01	7.30E+01	6.39E+01	1.45E+02	5.47E+02	**5.43E+01**
	Best	2.50E+03	2.50E+03	2.50E+03	2.52E+03	2.50E+03	2.50E+03	2.50E+03	2.53E+03	2.51E+03	**2.50E+03**
	Worst	2.65E+03	4.27E+03	3.28E+03	3.68E+03	2.66E+03	2.69E+03	2.65E+03	3.21E+03	4.46E+03	**2.63E+03**
F11	Mean	2.79E+03	2.86E+03	2.93E+03	3.36E+03	2.86E+03	2.99E+03	2.85E+03	3.68E+03	3.41E+03	**2.72E+03**
	Std	1.09E+02	1.73E+02	2.63E+02	**4.02E+01**	1.83E+02	2.62E+02	1.89E+02	4.45E+02	5.07E+02	1.16E+02
	Best	2.70E+03	2.73E+03	2.74E+03	3.21E+03	2.67E+03	2.79E+03	2.65E+03	2.93E+03	2.84E+03	**2.61E+03**
	Worst	3.24E+03	3.23E+03	3.93E+03	3.40E+03	3.34E+03	3.71E+03	3.23E+03	4.38E+03	4.57E+03	**3.14E+03**
F12	Mean	**2.87E+03**	2.93E+03	2.95E+03	3.08E+03	2.90E+03	2.96E+03	2.88E+03	3.12E+03	3.03E+03	2.87E+03
	Std	**7.61E+00**	8.23E+01	6.37E+01	3.09E+01	2.13E+01	3.52E+01	1.81E+01	7.29E+01	7.36E+01	1.97E+01
	Best	2.87E+03	2.87E+03	2.87E+03	2.99E+03	2.87E+03	2.91E+03	2.86E+03	3.00E+03	2.90E+03	**2.86E+03**
	Worst	**2.91E+03**	3.28E+03	3.15E+03	3.12E+03	2.94E+03	3.08E+03	2.92E+03	3.28E+03	3.21E+03	2.94E+03

## 5. Linear antenna array

### 5.1 Equally spaced linear array

The IERWHO algorithm is primarily used to determine the excitation amplitude of each element in a LAA. The design index for a system or device must consider various limitations to ensure optimal performance. These limitations include suppressing SLL, maintaining directionality, and placing zero points at specified positions. By addressing these limitations, designers can enhance the performance of the system and meet the desired requirements.

**Fig 7:**
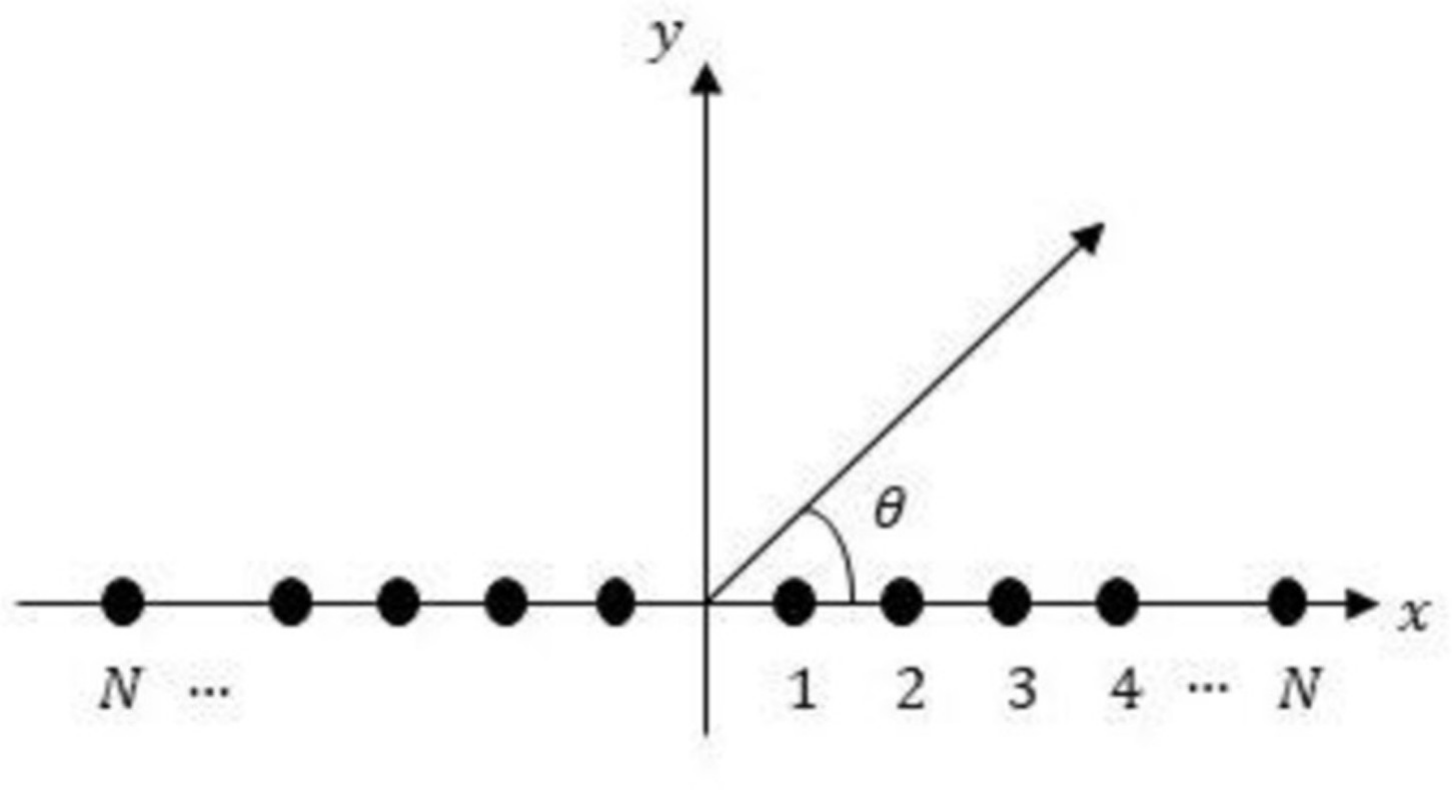
Uniform LAA geometry.

In this study, a LAA of 2*N* isotropic elements arranged symmetrically along *x* the axis is investigated, as depicted in Fig 7. Due to symmetry, the array factor (AF) in the azimuth plane is given by

$$\mathrm{AF}(\theta )=2\sum_{n=1}^{N}I_{n}\cos\left(kx_{n}\cos(\theta )+\psi_{n}\right)$$ (11)

where *k* = 2*π*/λ is the wave number and *I*_*n*_, *x*_*n*_, and $\psi_{n}$, respectively, represent the excitation amplitude, position, and phase of the nth element. *θ* symbolizes the scanning angle, which is defined as the angle with the positive x-axis. However, if it is further assumed that $\psi_{n}$ is zero, the array factor for only optimizing antenna current amplitude is given by Eq 12.


$$\mathrm{AF}(\theta )=2\sum_{n=1}^{N}I_{n}\cos\left(kx_{n}\cos(\theta )\right)$$ (12)

The objective is accomplished by selecting an appropriate fitness function, as follows:

Fitness=min(max(20log|AF(θ)|)) (13)

where *θ* ∈the spatial region in which SLL is suppressed AF(*θ*) is the array factor given by Eq 12.

The first experiment discussed in this passage focuses on the optimization of a 16-element LAA using IERWHO algorithm. The main objective of this optimization process is to effectively suppress the maximum SLL within the designated region, *θ* = [0°,80°] and *θ* = [100°,180°]. The IERWHO algorithm was independently executed 50 times employing MATLAB R2019b. The population number for the IERWHO algorithm is 20 and the maximum number of iterations is set at 500. The comparison of optimized amplitude excitation and antenna SLL optimized using different methods is shown in Table 7.

**Table 7:** Optimized current amplitudes for peak SLL minimization.

Method	Optimum results for the 16-element LAA	Maximum SLL (dB)
EFA [26]	1.0000, 0.9464, 0.8460, 0.7118, 0.5593, 0.4061, 0.2667, 0.2038	-33.62
WHO	1.0000, 0.9462, 0.8269, 0.7198, 0.5417, 0.4091, 0.2717, 0.1583	-32.10
IERWHO	1.0000, 0.9448, 0.8420, 0.7027, 0.5507, 0.3901, 0.2577, 0.1810	**-34.36**

**Fig 8:**
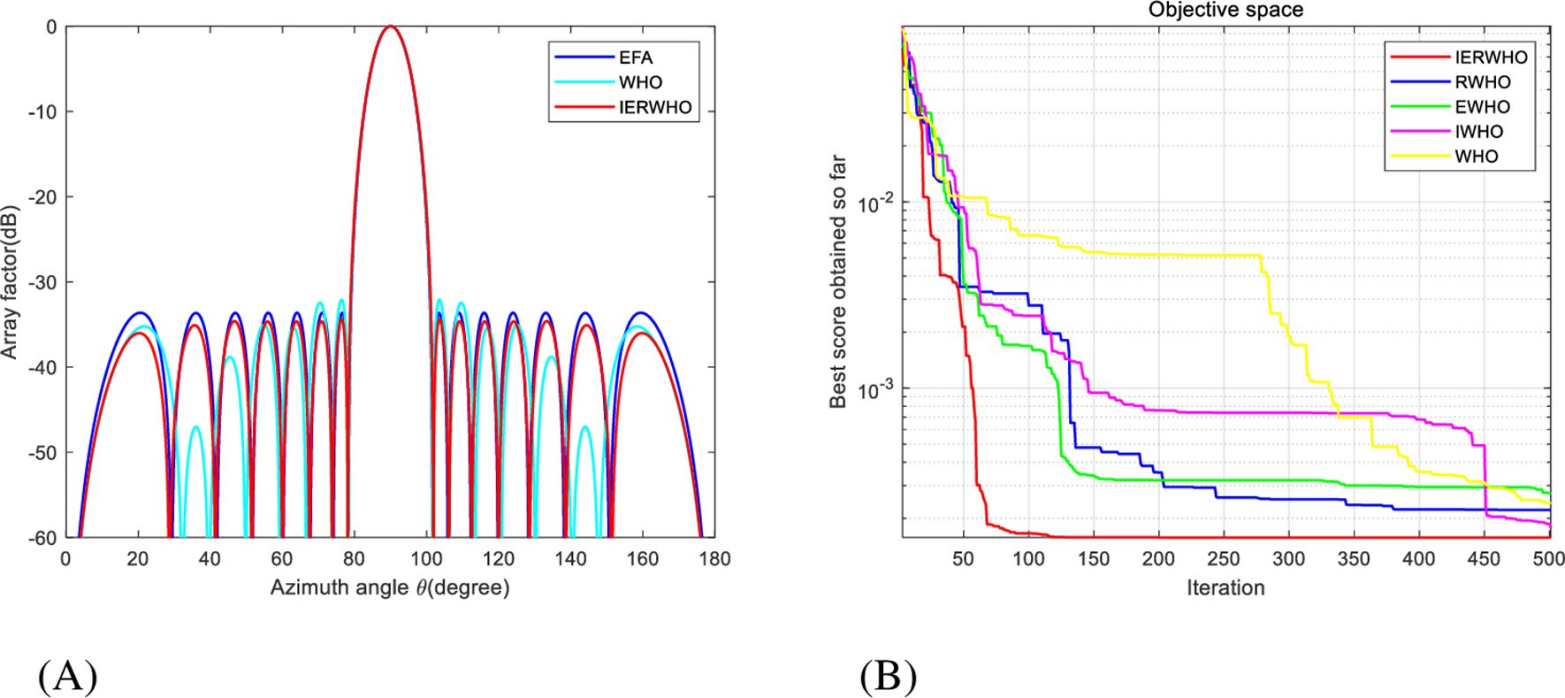
**A.** Radiation patterns of 16-element equally spaced LAA. **B.** Convergence plot of IERWHO algorithm for 16-element equally spaced LAA.

In experiment 1, the peak SLL obtained by the matrix optimized by IERWHO algorithm for experiment 1 is -34.36 dB, 0.74 dB less than the matrix optimized by EFA [26]. The peak SLL has been reduced from -32.10 dB to -34.36 dB (a decrease of 2.26 dB) compared to the WHO algorithm optimized array, as depicted in Table 7. Fig 8A and 8B showcase the beam pattern and convergence plot, respectively, exemplifying the remarkable efficacy of IERWHO algorithm in contrast to alternative algorithms.

The fitness function, as described in Eq 14, is employed to minimize SLL while also determining the optimal placement of nulls in the specified directions:

$$Fitness={\sum_{i}\frac{1}{\Delta \theta_{i}}\int_{\theta_{li}}^{\theta_{ui}}\left|AF\left(\theta \right)\right|}^{2}d\theta +{\sum_{k}\left|AF\left(\theta_{k}\right)\right|}^{2}$$ (14)

where *θ*_*ui*_ and *θ*_*li*_ are spatial regions in which SLL is suppressed and Δ*θ*_*i*_ = *θ*−*θ*_*li*_.The null direction is given by *θ*_*k*_. Eq 14 introduces a fitness function consisting of two terms. The first term focuses on restraining the SLL while the second term considers the positioning of nulls in the specified directions.*AF*(*θ*) is the array factor given by Eq 12.

Experiment 2 provides a linear array of 20-elements to achieve the minimizing of SLL in regions *θ* = [0°,82°] and *θ* = [98°,180°] as well as the placement of nulls at *θ* = 64°,76°,104°,*and* 116°. The population number for the IERWHO algorithm is 30 and the maximum number of iterations is set at 1000. Table 8 shows the optimized current amplitude obtained by using the IERWHO algorithm, WHO algorithm, GWO algorithm [53], and BBO algorithm [53].

**Table 8:** Optimized current amplitudes for peak SLL minimization.

Method	Optimum results for the 20-element LAA
BBO [53]	1.0000, 0.9747, 0.9264, 0.8575, 0.7022, 0.6242, 0.4799, 0.3607, 0.2369, 0.1234
GWO [53]	1.0000, 0.9794, 0.9254, 0.8126, 0.7008, 0.6000, 0.4594, 0.3326, 0.2133, 0.1167
WHO	1.0000, 0.9987, 0.9366, 0.8034, 0.7406, 0.5249, 0.4123, 0.3570, 0.2033, 0.0955
IERWHO	1.0000, 0.9922, 0.9881, 0.7832, 0.7134, 0.5480, 0.4318, 0.2932, 0.2105, 0.1438

**Fig 9:**
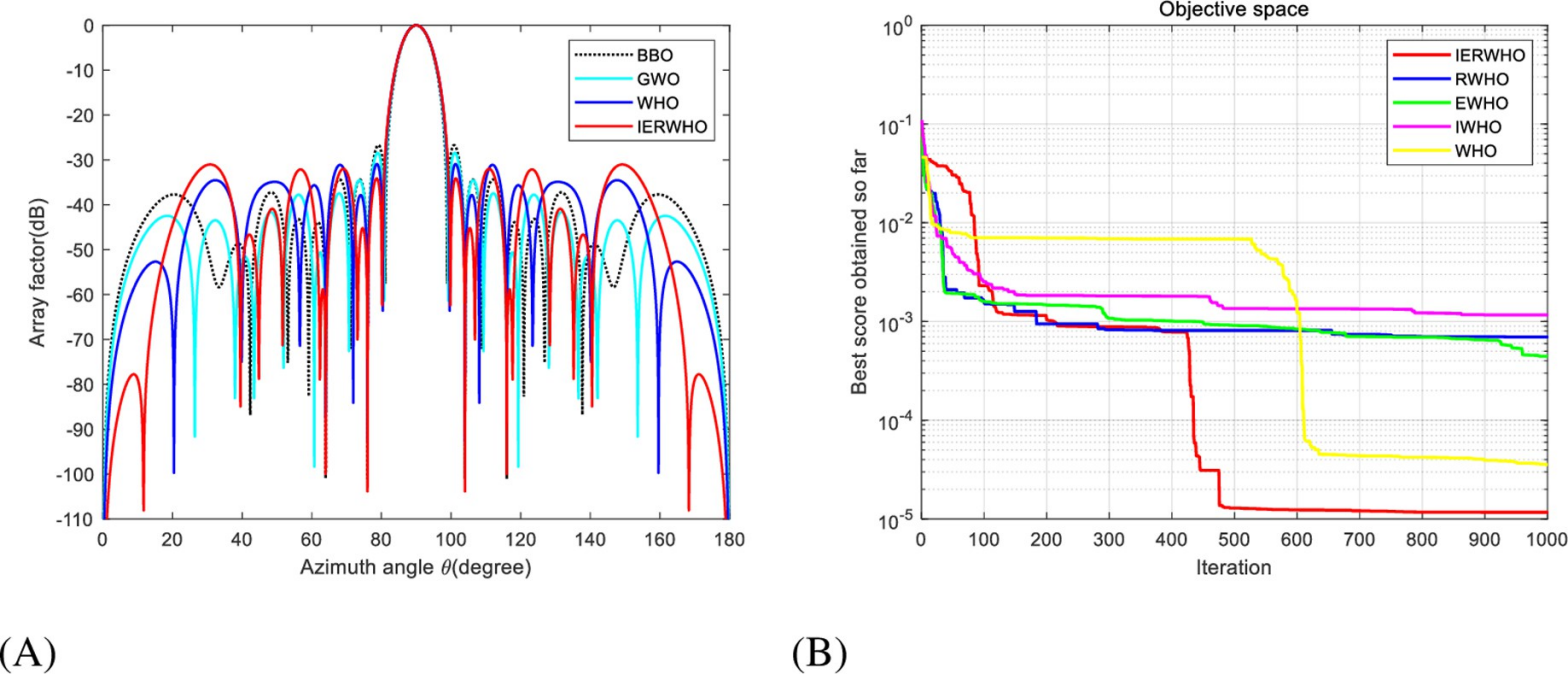
**A.** Radiation patterns of 20-element equally spaced LAA. **B.** Convergence plot of IERWHO algorithm for 20-element equally spaced LAA.

The IERWHO algorithm gives a peak SLL of -31.02 dB which is 4.31 dB lower as compared to the BBO algorithm array, 2.58 dB lower than the GWO array, and 0.09 dB lower than the WHO algorithm array. The optimized current amplitudes obtained using IERWHO, WHO, GWO, and BBO algorithm optimized arrays, are shown in Table 8, and the peak SLL and zero depth comparative analysis obtained are shown in Table 9. It is seen that the placement of null depth obtained by using the proposed approach is -112.3 dB which is32.64 dB lower than the BBO array,20.28dB lower than the GWO array, and 19.65 dB lower than the WHO algorithm array, as shown in Table 9. Fig 9A and 9B showcase the beam pattern and convergence plot, respectively, exemplifying the remarkable efficacy of IERWHO algorithm in contrast to alternative algorithms.

**Table 9:** Peak SLL and null depths for experiment 2.

Peak SLL (dB)			Null depth (dB)		
		64°	76°	104°	116°
BBO [53]	-26.71	-79.66	-74.2	-74.2	-79.66
GWO [53]	-28.44	-92.02	-79.12	-79.12	-92.02
WHO	-30.93	-87.73	-92.65	-92.65	-87.73
IERWHO	**-31.02**	**-112.3**	**-103.8**	**-103.8**	**-112.3**

### 5.2 Unequally spaced linear array

**Fig 10:**
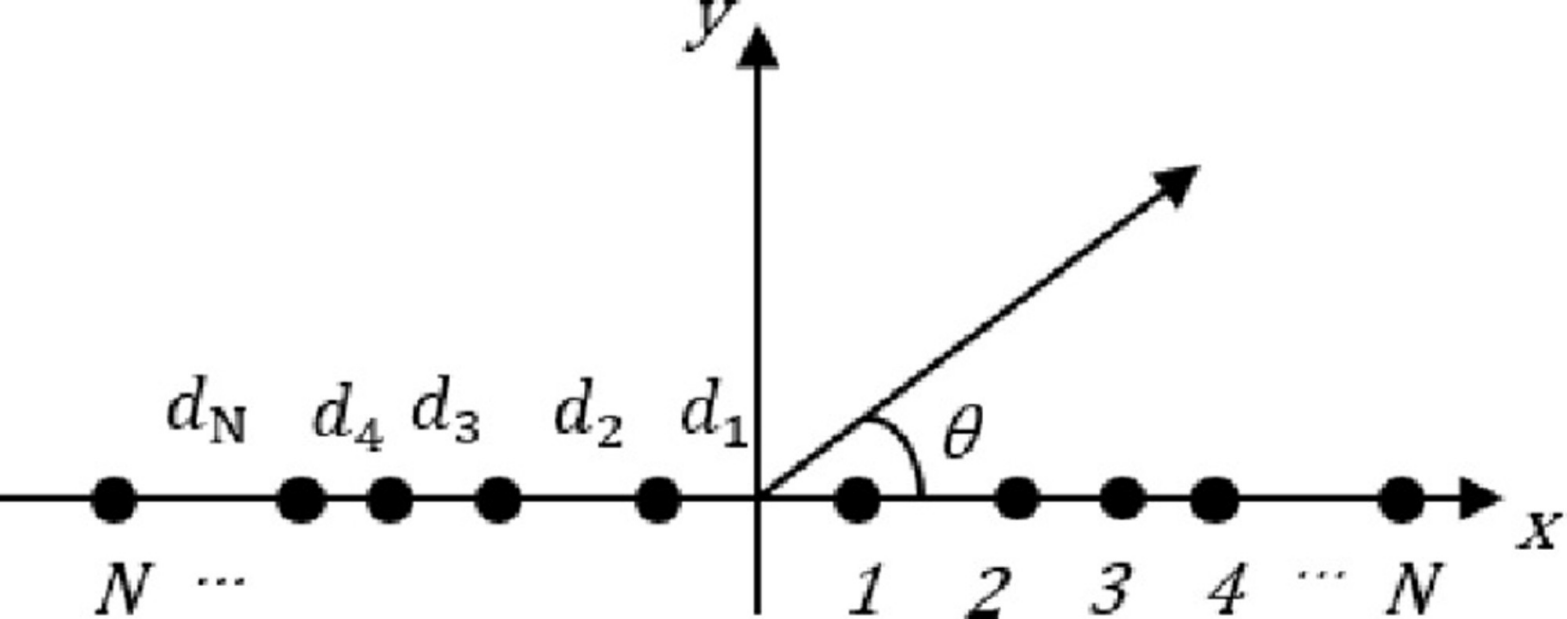
Sparse LAA geometry.

The separation between elements in a sparse LAA shown in Fig 10 is not uniform. The optimization guarantees that the space between elements always exceeds a certain distance threshold, while maintaining the amplitude and assuming the phases are 1 and 0 respectively. The sparse LAA array factor can be rewritten as:

$$AF(\theta )=2\sum_{n=1}^{N}\cos\left(kx_{n}\;\cos\theta \right)$$ (15)

In this section, optimization is focused on suppressing the maximum SLL by using element spacing and excitation amplitude as variables. Therefore, the fitness function can be formulated as

$$\text{min}\;\text{fitness}=\max\left\{20\log_{10}\frac{\left|AF\left(\theta_{SL}\right)\right|}{\left|\mathrm{AF}\left(\theta_{ML}\right)\right|}\right\},$$ (16)

$$\text{s.t}.\;\theta_{ML}=\text{arg}\;\text{max}|AF(\theta )|,\;\theta \in [0,\pi ],$$ (17)

$$\theta_{SL}\in \left[0,\theta_{FN1}\right]\cup \left[\theta_{FN2},\pi \right],$$ (18)

$$0<d_{i}<\lambda ,\;\forall i\in N,$$ (19)

$$0<I_{i}<1,\;\forall i\in N,$$ (20)

where *θ*_*SL*_ and *θ*_*ML*_ represent the region of the side lobe and main lobe, respectively. *θ*_*FN*1_ and *θ*_*FN*2_ are the first nulls of the pattern. The range of element spacing and excitation amplitude optimization is defined by constraints Eqs 19 and 20. The algorithm’s optimization ability is tested in different dimensions by selecting 16 and 28 elements. In order to maintain the first null beam width to the maximum extent, LAA with 2*N* of 16 and 28, *θ*_*FN*1_ is limited to 82°and 86°and *θ*_*FN*2_ is 98° and 94°, respectively.

In experiment 3, the entire length of the antenna array must be further restricted to preserve the main lobe’s shape and beam width, as demonstrated by the following formula:

$$\left\{\begin{array}{l} x_{1}=0.25\lambda ,\\ x_{N}=\frac{(2N-1)d}{2}. \end{array}\right.$$ (21)

To maintain Nyquist spatial sampling, the spacing of 0.5λ is established between the initial two elements on each side of the y-axis. This choice guarantees that one pair of antennas within the antenna array satisfies the criteria. Additionally, the Nth element remains fixed at *x*_*n*_ = (2*N*-1)d/2. Here, *d* represents the default spacing of the uniform LAA, set at 0.5*λ*. This specific configuration effectively mitigates any distortion of the main lobe. The positions of the remaining elements can be adjusted while the initial and final elements of the antenna array remain fixed in position. Therefore, the optimized dimension is simplified 2*N* - 2.

**Fig 11:**
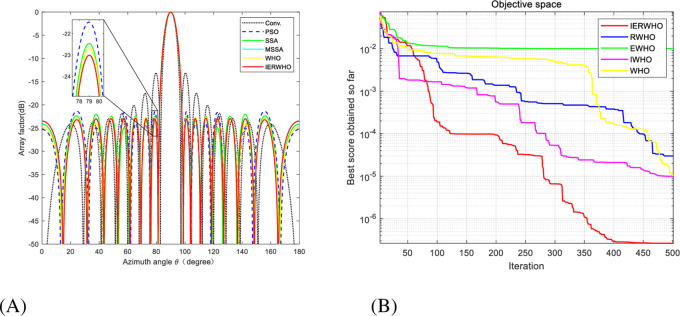
**A.** Radiation patterns of 16-element unequally spaced LAA. **B.** Convergence plot of IERWHO algorithm for 16-element unequally spaced LAA.

The IERWHO algorithm was independently executed 50 times employing MATLAB R2019b. The population number for the IERWHO algorithm is 30 and the maximum number of iterations is set at 500. The beam patterns of a 16-element LAA optimized by various algorithms are illustrated in Fig 11A. Fig 11B illustrates the iteration curves that correspond to the WHO, IWHO, EWHO, RWHO, and IERWHO algorithms. The beam patterns of a 16-element LAA optimized by various algorithms are illustrated in Fig 11A. Fig 11B illustrates the iteration curves that correspond to the WHO, IWHO, EWHO, RWHO, and IERWHO algorithms. Exemplifying the remarkable efficacy of IERWHO algorithm in contrast to alternative algorithms. The simultaneous display of Table 10 presents the optimized outcomes and the maximum SLL for a LAA with 16 elements, employing diverse algorithms. Compared with the conventional method, PSO, SSA, MSSA [54], WHO, and IERWHO algorithms has a better optimization effect, and the maximum SLL is reduced by 9.78 dB, 1.56 dB, 0.91 dB, 0.25 dB, and 0.33 dB, respectively.

**Table 10:** Peak SLL and null depths for experiment 3.

Method	Optimized element positions(λ)	Maximum SLL (dB)
Conv.	0.2500, 0.7500, 1.2500, 1.7500, 2.2500, 2.7500, 3.2500, 3.7500	-13.15
PSO [54]	0.2500, 0.5311, 1.0128, 1.3930, 1.8738, 2.3329, 2.9893, 3.7500	-21.37
SSA [54]	0.2500, 0.5331, 1.0118, 1.3453, 1.8495, 2.3404, 2.9835, 3.7500	-22.02
MSSA [54]	0.2500, 0.5226, 1.0038, 1.3486, 1.8518, 2.3447, 2.9948, 3.7500	-22.68
WHO	0.2500, 0.5209, 1.0003, 1.3560, 1.8522, 2.3392, 3.0027, 3.7500	-22.60
IERWHO	0.2500, 0.5123, 1.0017, 1.3458, 1.8452, 2.3264, 2.9879, 3.7500	**-22.93**

**Fig 12:**
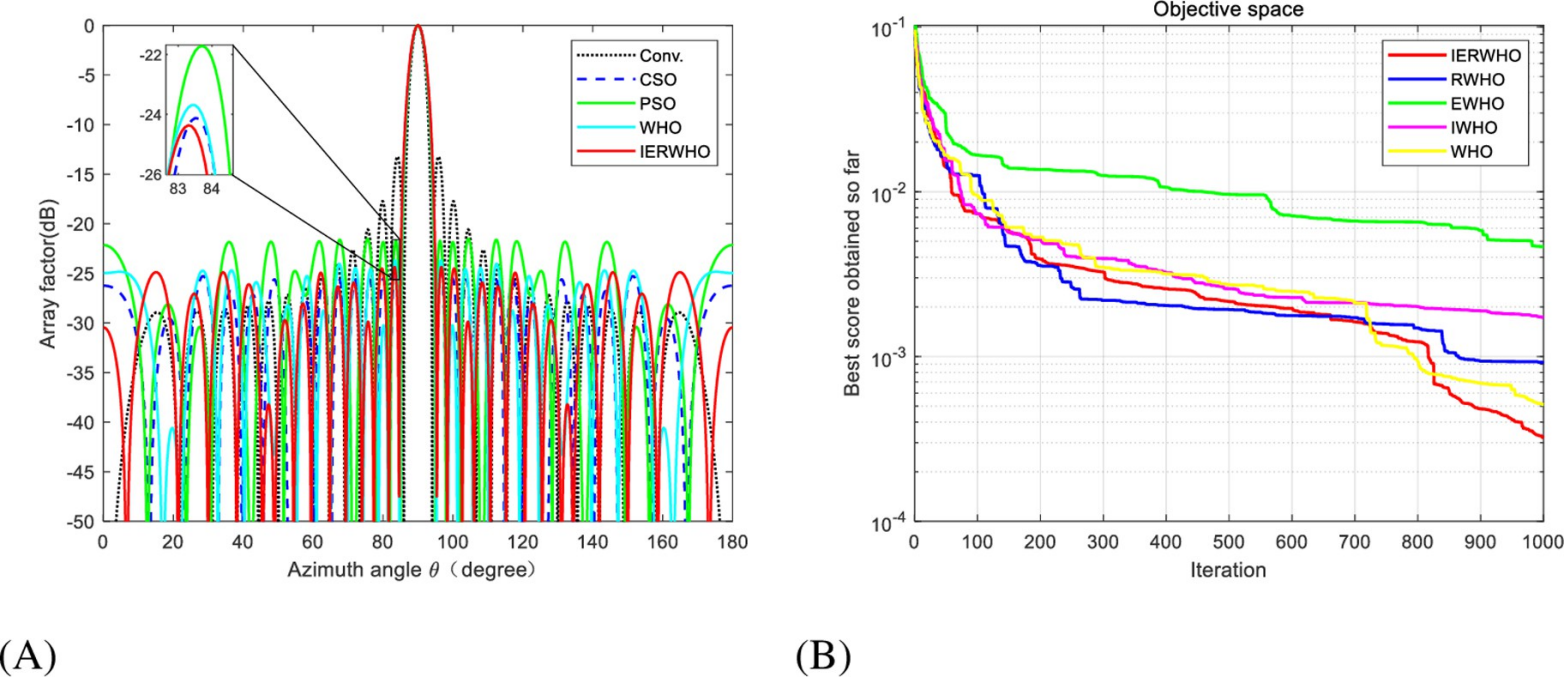
**A**. Radiation patterns of 28-element unequally spaced LAA. **B**. Convergence plot of IERWHO algorithm for 28-element unequally spaced LAA.

In experiment 4, The population number for the IERWHO algorithm is 50 and the maximum number of iterations is set at 1000. The beam patterns of a 28-element LAA optimized by various algorithms are illustrated in Fig 12A. Fig 12B illustrates the iteration curves that correspond to the WHO, IWHO, EWHO, RWHO, and IERWHO algorithms. Fig 12A and 12B showcase the beam pattern and convergence plot, respectively, exemplifying the remarkable efficacy of IERWHO algorithm in contrast to alternative algorithms. The simultaneous display of Table 11 presents the optimized outcomes and the maximum SLL for a LAA with 28 elements, employing diverse algorithms. Compared with the conventional method, CSO [55], PSO, WHO, and IERWHO algorithm has a better optimization effect, and the maximum SLL is reduced by 11.28 dB, 0.38 dB, 3.02 dB, and 0.5 dB, respectively.

**Table 11:** Peak SLL and null depths for experiment 4.

Method	Optimized element positions(λ)	Maximum SLL (dB)
Conv.	0.2500, 0.7500, 1.2500, 1.7500, 2.2500, 2.7500, 3.2500, 3.7500, 4.2500, 4.7500, 5.2500, 5.7500,6.2500,6.7500	-13.23
CSO [55]	0.2344, 0.5280, 0.9224, 1.2965, 1.6549, 2.1427, 2.4387, 2.9369, 3.3753, 3.9280, 4.4091, 5.1167, 5.9188, 6.7422	-24.13
PSO [55]	0.1703, 0.6430, 0.9509, 1.4245, 1.7849, 2.0397, 2.4511, 3.0522, 3.6249, 4.0476, 4.6302, 5.2984, 5.9582, 6.7118	-21.49
WHO	0.2500, 0.5297, 0.9541, 1.4107, 1.5678, 2.2188, 2.3857, 3.0639, 3.2210, 3.8887, 4.3468, 5.0500, 5.9226, 6.7497	-24.01
IERWHO	0.2515, 0.4042, 1.0191, 1.0728, 1.7286, 1.8898, 2.4694, 2.8092, 3.3002, 3.8023, 4.2946, 4.9019, 5.7096, 6.5482	**-24.51**

Amplitudes of 16-, 20-element equally spaced LAA and positions of 16-, 28-element unequally spaced LAA using IERWHO algorithm method to achieve the optimal design. Table 12 shows the comparative result of the algorithm in terms of best cost (dB), worst cost (dB), the mean cost (dB), and standard deviation for the 50 runs.

**Table 12:** Performance of the IERWHO algorithm in 50 runs of four LAA models.

Case	Best	Worst	Mean	Std
Experiment 1	-34.36	-16.69	-30.46	4.29
Experiment 2	-31.02	-12.32	-26.25	4.23
Experiment 3	-22.93	-15.38	-21.67	1.92
Experiment 4	-24.51	-12.64	-18.61	3.04

## 6. Conclusion

This paper proposes an IERWHO algorithm to address the insufficiency of WHO algorithm in solving practical problems. The algorithm incorporates three strategies: including a logistic-tent sequence during the population initialization stage; modifying the adaptive parameter TDR, which is common in similar algorithms, into a nonlinear factor; and adding the random inertia weight to the water competition mechanism. The effectiveness of each strategy was evaluated by conducting experiments on a set of 12 benchmark test functions. According to research, the search efficiency and stability of IERWHO algorithm are enhanced, while also improving the local search capability and optimizing accuracy when contrasted with WHO, IWHO, EWHO, and RWHO algorithms. The optimization results of IERWHO algorithm and the swarm intelligence optimization algorithms were evaluated by using the CEC2022 test function. The simulation findings demonstrate that IERWHO algorithm outperforms other algorithms across a majority of the test functions, thereby substantiating the efficacy of IERWHO algorithm.

Four optimization problems of antenna array pattern synthesis are proposed and solved using IERWHO algorithm in this paper. Simulation results on 16 symmetric LAA show that IERWHO algorithm has superior performance compared with EFA and WHO algorithms. IERWHO algorithm achieves a peak SLL of -34.36 dB when synthesizing the amplitude current of 16-element LAA. When comparing IERWHO algorithm with BBO, GWO, and WHO algorithms, the simulation results exhibit outstanding performance in the synthesis of 20-element LAA. By utilizing IERWHO algorithm, the amplitude optimization of a 20-element LAA was accomplished with a null depth of -112.3 dB and a peak SLL of -31.02 dB. Additionally, it attains a peak SLL of -22.93 dB and -24.51 dB when synthesizing sparse 16 and 28 LAA respectively. Compared to the conventional method, PSO, CSO, SSA, MSSA, and WHO algorithms, these results are superior. In summary, significant outcomes are obtained for linear configurations with different constraints. The findings of the IERWHO algorithm study underscore its efficacy in addressing intricate antenna design challenges, thereby showcasing its potential to offer valuable insights for resolving other practical applications.

Although the current set of antenna models has shown that the IERWHO algorithm is effective, there may be limitations in its scalability to larger or more intricate antenna systems. Different challenges may arise when trying to extend the algorithm’s effectiveness to other types of antenna arrays or various optimization problems. Future research should explore the potential of IERWHO algorithm in complex, high-dimensional real-world applications, including its use in training multilayer perceptron for classification tasks. Additionally, by combining IERWHO algorithm with other meta-heuristic algorithms, there is an opportunity to enhance its optimization performance. This hybrid approach could be particularly advantageous when addressing diverse optimization challenges, such as optimizing concentric ring and elliptical antenna arrays, feature selection, multilevel thresholding image segmentation, and parameter optimization.

## Supporting information

S1 File (DOCX)
