# Supplementary material for: A Wild Horse Optimization algorithm with chaotic inertia weights and its application in linear antenna array synthesis
Source: PLoS One. 2024 Jul 5;19(7):e0304971. doi: 10.1371/journal.pone.0304971 (PMC11226036; doi:10.1371/journal.pone.0304971)
Supplement: S1 File — (DOCX) [file pone.0304971.s001.docx]

Dear Editor,

Thank you for your prompt response and for bringing this matter to my attention. I apologize for the oversight regarding the inclusion of the Supporting Information files with my submission.

I will ensure that the minimal data set is promptly uploaded as a Supporting Information file to accompany the manuscript. Additionally, I will provide separate captions for the supplementary files as per your instructions.

Thank you for your understanding.

Best regards,

<Wanru Zhao> et al.

Supplementary files

The comparison data for each of the four examples of optimized linear antenna arrays in the paper are from published papers, as follows:

Example 1 is a comparison with the 16-element array LAA optimization in the paper "[Synthesis of Linear Antenna Arrays Using Enhanced Fireﬂy Algorithm Spaced](https://link.springer.com/article/10.1007/s13369-018-3214-2)" by Urvinder Singh and Rohit Salgotra.

Example 2 is compared to the Example 7 LAA in the paper "[Optimal Pattern Synthesis of Linear Antenna Array Using Grey Wolf Optimization Algorithm](https://www.hindawi.com/journals/ijap/2016/1205970/)" by Prerna Saxena and Ashwin Kothari Optimization for comparison.

Example 3 is a comparison with the paper published by Qiankun Liang ,Bin Chen, Huaning Wu, "[A Novel Modified Sparrow Search Algorithm with Application in Side Lobe Level Reduction of Linear Antenna Array](https://www.hindawi.com/journals/wcmc/2021/9915420/)" for comparison with the 16-element array LAA optimization.

Example 4 is compared with Example 4 LAA optimization in the paper "[Linear Antenna Array Synthesis Using Cat Swarm Optimization](https://www.sciencedirect.com/science/article/abs/pii/S1434841113003208)" by Lakshman Pappula, Debalina Ghosh.

The array antenna beam pattern for the above four examples in the manuscript, Fig 8A, Fig 9A, Fig 11A, and Fig 12A, are drawn from the data in Tables 7, 9, 10, and 11, respectively.

We are sorry that the chaotic inertia-weighted Wild Horse optimization (IERWHO) optimized linear array proposed in this paper is protected by a patent and the exact code cannot be provided. The patent number is: 202310914077.9. The table below provides the maximum sidelobe levels obtained by repeating the algorithm 50 times for four examples.

**Table 1. The maximum sidelobe levels obtained by repeating the algorithm 50 times for four examples.**

|  | **Experiment 1** | **Experiment 2** | **Experiment 3** | **Experiment 4** |
| --- | --- | --- | --- | --- |
| **Maximum** **SLL of 50 runs (dB)** | -32.534 | -26.518 | -22.859 | -14.499 |
|  | -32.341 | -26.016 | -22.814 | -20.552 |
|  | -32.854 | -24.476 | -22.827 | -18.176 |
|  | -22.596 | -25.188 | -22.172 | -13.976 |
|  | -31.249 | -30.013 | -22.445 | -17.686 |
|  | -32.237 | -27.782 | -22.887 | -19.045 |
|  | -31.642 | -29.68 | -22.449 | -17.686 |
|  | -32.293 | -29.291 | -22.849 | -23.766 |
|  | -32.15 | -18.088 | -22.874 | -23.918 |
|  | -32.197 | -26.444 | -22.756 | -23.453 |
|  | -32.027 | -18.217 | -22.347 | -18.978 |
|  | -31.556 | -28.885 | -21.827 | -19.259 |
|  | -19.165 | -28.97 | -22.877 | -20.067 |
|  | -32.254 | -12.317 | -22.667 | -14.874 |
|  | -32.634 | -17.659 | -22.76 | -16.698 |
|  | -31.849 | -29.938 | -20.516 | -20.695 |
|  | -30.454 | -28.907 | -22.878 | -15.642 |
|  | -32.727 | -25.657 | -22.416 | -17.839 |
|  | -27.078 | -23.708 | -22.463 | -17.921 |
|  | -20.383 | -26.49 | -22.716 | -24.439 |
|  | -32.969 | -25.485 | -22.324 | -19.901 |
|  | -31.338 | -18.765 | -22.584 | -22.149 |
|  | -31.843 | -24.871 | -16.438 | -23.174 |
|  | -32.842 | -25.267 | -18.404 | -17.44 |
|  | -21.778 | -28.531 | -22.121 | -14.829 |
|  | -32.595 | -30.97 | -22.626 | -17.212 |
|  | -32.452 | -20.482 | -22.483 | -16.632 |
|  | -34.36 | -29.353 | -22.759 | -22.259 |
|  | -32.404 | -30.149 | -22.497 | -19.56 |
|  | -31.96 | -26.658 | -22.883 | -12.979 |
|  | -32.64 | -21.389 | -19.602 | -21.14 |
|  | -32.519 | -30.074 | -22.934 | -13.499 |
|  | -32.512 | -23.515 | -22.861 | -18.08 |
|  | -32.816 | -26.262 | -21.986 | -16.803 |
|  | -21.105 | -28.658 | -21.636 | -19.414 |
|  | -32.423 | -30.461 | -22.366 | -22.566 |
|  | -32.678 | -28.156 | -17.062 | -19.264 |
|  | -32.978 | -25.293 | -22.78 | -17.609 |
|  | -16.692 | -29.255 | -18.786 | -21.992 |
|  | -32.146 | -27.206 | -19.226 | -13.086 |
|  | -32.543 | -27.056 | -22.86 | -18.173 |
|  | -32.36 | -30.244 | -22.803 | -18.377 |
|  | -32.662 | -27.116 | -17.823 | -18.474 |
|  | -32.399 | -30.592 | -22.848 | -22.158 |
|  | -30.896 | -30.097 | -21.689 | -18.463 |
|  | -21.431 | -17.357 | -22.338 | -18.292 |
|  | -32.765 | -27.064 | -22.842 | -20.344 |
|  | -27.639 | -30.811 | -21.993 | -12.635 |
|  | -32.474 | -27.287 | -18.349 | -19.296 |
|  | -32.358 | -29.717 | -15.378 | -15.477 |
| **Best (dB)** | **-34.36** | **-31.02** | **-22.934** | **-24.51** |
| **Worst (dB)** | **-16.692** | **-12.317** | **-15.378** | **-12.635** |
| **Mean (dB)** | **-30.456** | **-26.249** | **-21.674** | **-18.61** |
| **Std (dB)** | **4.2901** | **4.2306** | **1.9204** | **3.0371** |

Below are the relevant links to the algorithm testing paper and code. If you need the code for other metaheuristics algorithms mentioned in the paper, you can request it from the author.

The WHO algorithm references mentioned in this paper are "[Wild horse optimizer: a new meta‑heuristic algorithm for solving engineering optimization problems](https://link.springer.com/article/10.1007/s00366-021-01438-z)". The code for the WHO algorithm can be accessed on the MATLAB Central File Exchange platform at the following link: [Wild Horse Optimizer - File Exchange - MATLAB Central (mathworks.cn)](https://ww2.mathworks.cn/matlabcentral/fileexchange/90787-wild-horse-optimizer).

The test functions in section 4.1 refer to two papers as "[Wild horse optimizer: a new meta-heuristic algorithm for solving engineering optimization problems](https://link.springer.com/article/10.1007/s00366-021-01438-z)" and "[An exploration-enhanced grey wolf optimizer to solve high-dimensional numerical optimization](https://www.sciencedirect.com/science/article/abs/pii/S0952197617302713)". In order to make the test functions richer and show the performance of the algorithms more comprehensively.

The testing functions for section 4.2 can be found in the CEC2022 code, accessible on the MATLAB Central File Exchange platform at the following link: [CEC2022 - File Exchange - MATLAB Central (mathworks.cn)](https://ww2.mathworks.cn/matlabcentral/fileexchange/157611-cec2022?s_tid=srchtitle_site_search_1_CEC2022).
